# Regulatory T cells in tumor microenvironment: new mechanisms, potential therapeutic strategies and future prospects

**DOI:** 10.1186/s12943-020-01234-1

**Published:** 2020-07-17

**Authors:** Chunxiao Li, Ping Jiang, Shuhua Wei, Xiaofei Xu, Junjie Wang

**Affiliations:** 1 Department of Radiation Oncology, Peking University Third Hospital, Beijing, 100191 China; 2 Center for Reproductive Medicine, Department of Obstetrics and Gynecology, Peking University Third Hospital, Beijing, 100191 China; 3 National Clinical Research Center for Obstetrics and Gynecology, Key Laboratory of Assisted Reproduction, Ministry of Education, Peking University, Beijing, 100191 China

## Abstract

Regulatory T cells (Tregs) characterized by the expression of the master transcription factor forkhead box protein p3 (Foxp3) suppress anticancer immunity, thereby hindering protective immunosurveillance of tumours and hampering effective antitumour immune responses in tumour-bearing hosts, constitute a current research hotspot in the field. However, Tregs are also essential for the maintenance of the immune tolerance of the body and share many molecular signalling pathways with conventional T cells, including cytotoxic T cells, the primary mediators of tumour immunity. Hence, the inability to specifically target and neutralize Tregs in the tumour microenvironment without globally compromising self-tolerance poses a significant challenge. Here, we review recent advances in characterizing tumour-infiltrating Tregs with a focus on the functional roles of costimulatory and inhibitory receptors in Tregs, evaluate their potential as clinical targets, and systematically summarize their roles in potential treatment strategies. Also, we propose modalities to integrate our increasing knowledge on Tregs phenotype and function for the rational design of checkpoint inhibitor-based combination therapies. Finally, we propose possible treatment strategies that can be used to develop Treg-targeted therapies.

## Introduction

Regulatory T cells (Tregs), as an important mechanism for regulating homeostasis of the immune system and the immune tolerance of the body, play crucial roles in the regulation of tumour immunity and constitute a current research hotspot in the field, primarily as potential targets (Supplementary Table 1) that can inhibit the activation and differentiation of CD4^+^ helper T cells and CD8^+^ cytotoxic T cells to induce reactivity against autologous and tumour-expressed antigens [1–3]. In the tumour microenvironment (TME), Tregs can be induced and differentiated by traditional T cells, which have a strong immunosuppressive function, inhibit antitumour immunity, and promote the occurrence and development of tumours. Tregs can also suppress the function of immune effector cells through a variety of mechanisms and are key factors in tumour immune escape [4–7].

In the early 1970s, the concept of suppressor T cells was clearly proposed [8–10], and in 1975, some scholars speculated that suppressor T cells might be closely related to the occurrence and development of tumours. It was not until 1980 that researchers confirmed the presence of suppressor T cells in a series of studies [11]. In 1990, suppressor T cell cloning was successfully performed for the first time, which confirmed the existence of suppressor T cells against tumour immunity in vivo [12, 13]. In 1995, Sakaguchi et al. found that the binding chain of the IL-2 receptor, namely, the CD25 molecule, can be used as a surface marker of suppressor T cells, and the concept of Tregs was clearly proposed [14, 15]. However, later, Shimon Sakaguchi et al. found that forkhead/winged helix transcription factor (Foxp3) was specifically expressed in Tregs, and CD4^+^CD25^+^Foxp3^+^ is currently considered to be a classical combined marker of Tregs [16, 17]. In fact, in addition to its ability to label Tregs, Foxp3 dominantly controls Tregs function, and only its continuous expression guarantees the maintenance of full Tregs suppressive capacity [18–23]. Although Foxp3 is a transcription factor, its exact function remains largely unknown. It has been suggested that Foxp3 may act as a repressor of transcription upon activation [24]. It has also been proposed that all human CD4^+^ and CD8^+^ T cells may upregulate Foxp3 and acquire suppressive properties upon activation [25–27]. It was also found that the number of local Tregs in tumours was closely related to the progression and prognosis of tumours, and it was found to be a good reference index for tumour prognosis [28]. The specific elimination of Tregs in vivo can effectively stimulate the antitumour immune response of tumour patients. Since 2006, the role of Tregs in tumour immunity and their mechanisms have been further studied. In the process of tumour immune escape, Tregs can secrete TGF-β, IL-10, and IL-35 (Ebi3-IL-12α heterodimer) [29], which downregulate antitumour immunity, suppress antigen presentation by DCs, CD4^+^ T helper (Th) cell function and generate tumour-specific CD8^+^ cytotoxic T lymphocytes (CTLs). The cytokines IL-10 and IL-35 are divergently expressed by Tregs subpopulations in the TME and synergistically promote intratumoural T cell exhaustion by regulating the expression of several inhibitory receptors and the exhaustion-associated transcriptomic signatures of CD8^+^ TILs [30]. The other Tregs functions include direct destruction of other cells by secreting perforin and granzyme and the synthesis and release of cyclic adenosine phosphate (cAMP) to interfere with the metabolism of other cells. As research has progressed, researchers proposed removing the inhibition of Tregs by clearing them, but there were problems with this approach. To address these problems, researchers proposed a new strategy for controlling, but not eliminating Tregs.

## Classifications and functions of Tregs

**Fig. 1:**
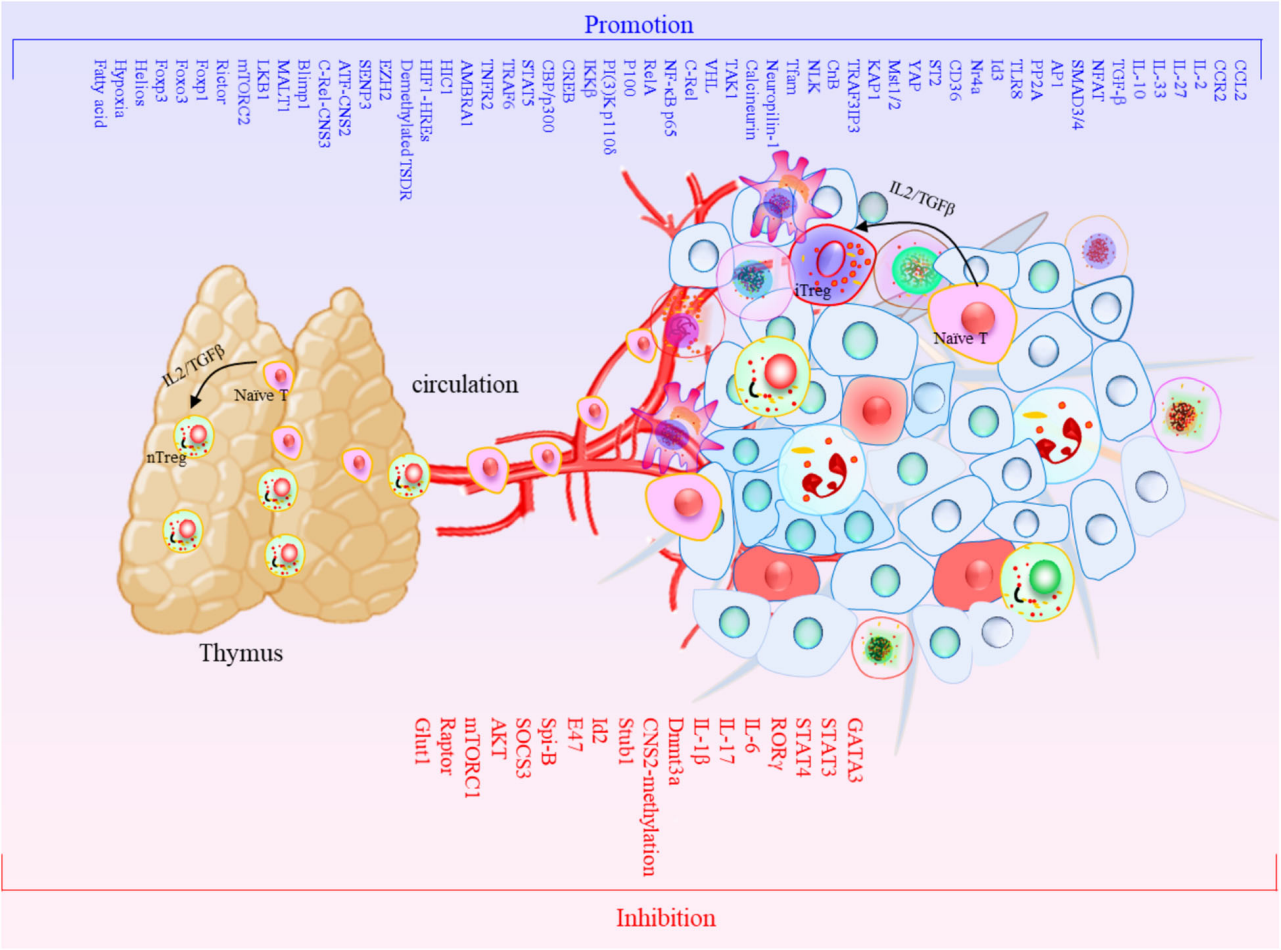
The differentiation of Tregs in tumor from naïve T cell in thymus through circulation. Treg is derived from peripheral naive T cells induced by tumor microenvironmental signals, including tumor antigens, cytokines (such as TGF-β) and some metabolic factor, which are concluded now. Furthermore, these promotion and inhibition factors are explained in this review

**Fig. 2:**
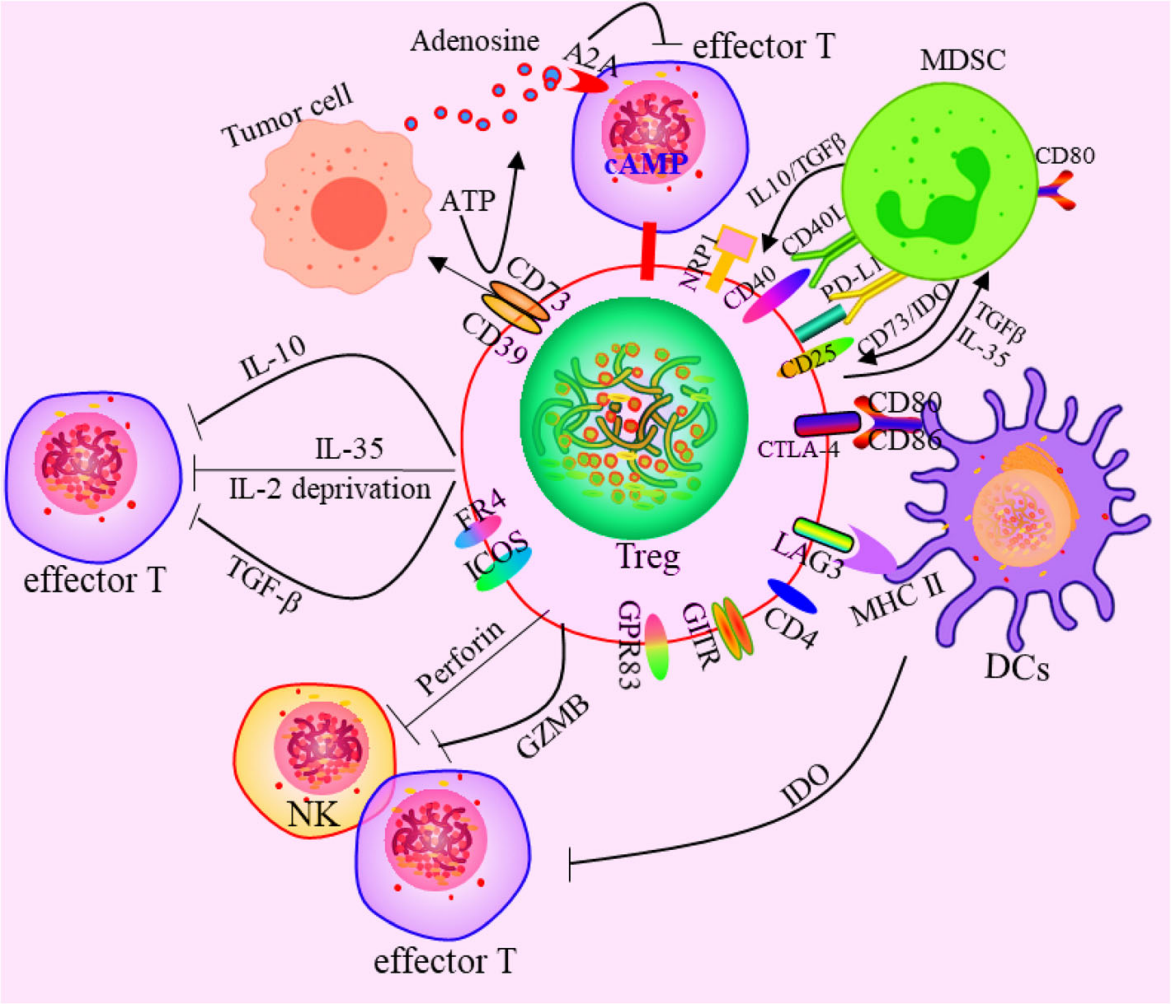
Effects of Tregs on the immune cells. The mechanism mainly includes four aspects:①secreting inhibitory cytokines, including IL-10, TGF-β, IL-35 etc., eg. inhibiting immune function through il-10 and other dependent ways, and Treg can also inhibit CD8+ T cells and DCs through membrane-bound TGF-β, thereby regulating the body‘s anti-tumor immune function. ②killing effector cells by granulase and perforin. Granzyme and perforin are the main molecules that mediate the cytotoxicity of CTL, NK and other cells.③Tregs affect effector cell function by interfering with cell metabolism mainly in the following three ways:(1) Deprivation of IL-2 in the TME, and the growth of Tregs and effector T cells requires the maintenance of IL-2. (2) CD39 and CD73 are nucleases that are constitutionally expressed in human and mouse Tregs. They can hydrolyse extracellular ATP or ADP into AMP and produce Adenosine. Tregs promotes the production of adenosine in the TME by producing the extracellular enzymes CD39 and CD73, and produces inhibitory and anti-proliferative effects by binding to the adenosine receptor A2A on the surface of effector cells. (3) Treg transferred a large number of cAMP to effector T cells through gap junction to interfere with their metabolism. ④Affect the differentiation and proliferation of Tregs by regulating DCs. The Tregs-expressed CTLA-4 was combined with CD80 and CD86 on the surface of DCs to downregulate the synergistic stimulus signal. Lymphocyte activation gene 3 (LAG3) molecules expressed by Tregs can inhibit the expression of MHC II molecules in DCs. DCs tolerance can be induced by the above two methods, and the latter can further induce T cell incapacity by IDOc. ⑤There exist the functional crosstalk between Tregs and MDSCs. Factors produced by both MDSCs and Tregs form positive feedback loops to facilitate the expansion of each population and reinforce the suppressive environment. On the one hand, MDSCs promoted the induction of Tregs through producing molecules including TGF-β, IL10, CD73, and IDO. On the other hand, Tregs can also modulate MDSCs expansion and function through secreting IL-35 and TGF-β. Additionally, cell-surface molecular interactions can promote the function of both MDSC and Tregs, including CD40/CD40L, PD-1/PD-L1, and CD80/CTLA-4

According to their products and biological characteristics, Tregs can be divided into two groups: natural regulatory T cells (naturally occurring Tregs, nTregs) and induced-to-adjust T cells (inducible Tregs, iTregs). Both types of Tregs can universally express Foxp3 [31–33]. nTregs develop naturally in the thymus, and their inhibitory effect is achieved through intercellular contact. Their main function is to maintain normal immune tolerance and control the inflammatory response, which can be activated and stabilized by NF-κB [34–36]. Thymic nTregs are generated through two distinct developmental programmes involving CD25^+^ Treg progenitors (CD25^+^ TregP cells) and Foxp3^lo^ Treg progenitors (Foxp3^lo^ TregP cells). CD25^+^ TregP cells show higher rates of apoptosis and interact with self-antigens with higher affinity than do Foxp3^lo^ TregP cells and have a TCR repertoire and transcriptome distinct from that of Foxp3^lo^ TregP cells. CD25^+^ TregP cells and Foxp3^lo^ TregP cells originate by acquiring negative-selection programmes and positive-selection programmes, respectively [37]. iTregs are derived from peripheral naive T cells induced by tumour microenvironmental signals, including tumour antigens, cytokines (such as TGF-β) and other soluble molecules [38] (Fig. 1). The TCR repertoires of tumour-resident iTregs vary yet display significant overlap with circulating Tregs. TCRs isolated from Tregs display specific reactivity against autologous tumours and mutated neoantigens, suggesting that intratumoural Tregs act in a tumour antigen-selective manner, leading to their activation and expansion in the TME. Tumour antigen-specific Treg-derived TCRs reside in the tumour and in the circulation, suggesting that both Tregs types serve as sources for tumour-specific TCRs [39]. Th17 cells are sources of tumour-induced Foxp3^+^ cells. In addition to nTregs and iTregs that develop from naive precursors, suppressive IL-17A^+^Foxp3^+^ and ex-Th17 Foxp3^+^ Tregs are sources of tumour-associated iTregs [40]. In nonlymphoid tissue, single-cell RNA-sequencing (scRNA-seq) was used to identify two precursor stages of IL-33 receptor ST2-expressing nonlymphoid tissue Tregs, residing in the spleen and lymph nodes. Global chromatin profiling of nonlymphoid tissue Tregs and two precursor stages revealed the stepwise acquisition of chromatin accessibility and reprogramming towards the nonlymphoid-tissue Tregs phenotype [41]. iTregs can be negatively regulated by the homeobox protein Hhex through the inhibition of Foxp3 expression and function. Furthermore, Hhex expression is significantly repressed in Tregs by TGF-β/Smad3 signalling [42]. iTregs inhibit the antitumour immune action of effector T cells (Teff), NK cells and DCs through a variety of mechanisms that promote tumour progression. Besides, there also exist the functional crosstalk between Tregs and MDSCs. The following five main mechanisms characterize Tregs (Fig. 2): ①Tregs secrete inhibitory cytokines, including IL-10, TGF-β, and IL-35, inhibiting immune function through IL-10 and other dependent pathways, and Tregs can inhibit CD8^+^ T cell and DC function through membrane-bound TGF-β, thereby regulating the body’s antitumour immune function [43–46]. ②Tregs kill effector cells by granzymes and perforin, the main molecules that mediate the cytotoxicity of CTL, NK and other cells. nTregs can kill target cells through granzyme A (GzmA) and perforin. nTregs do not produce granzyme B (GzmB), while iTregs highly express GzmB. Additionally, Tregs orchestrate memory T cell quiescence by repressing effector and proliferation programmes through the inhibitory receptor CTLA-4. Loss of Tregs leads to the activation of effector T cells in a genome-wide transcription programme characterized by the transition to and the establishment of memory CD8^+^ T cells for the terminal differentiation of the KLRG1^hi^ IL-7Rα^lo^ GzmB^hi^ phenotype with compromised metabolism affecting fitness, longevity, polyfunctionality, and protective efficacy. CTLA-4 functionally replaces Tregs in trans to reverse memory T cell defects and restore homeostasis [47]. Canonical NK cells are highly susceptible to Treg-mediated suppression, in contrast to the highly resistant CD57^+^ FcεRγ-NKG2C^+^ adaptive (CD56^+^CD3^−^) NK cells, which expand in cytomegalovirus-exposed individuals. Specifically, Tregs suppress canonical but not adaptive NK proliferation, IFN-γ production, degranulation, and cytotoxicity. Treg-mediated suppression is associated with canonical NK downregulation of TIM-3 and upregulation of the NK inhibitory receptors PD-1 and the IL-1R family member IL-1R8. Tregs production of the IL-1R8 ligand IL-37 contributes to phenotypic changes and diminishes the function of Treg-suppressed canonical NK cells. Blocking PD-1, IL-1R8, or IL-37 abrogates Tregs suppression of canonical NK cell functions while maintaining NK-cell TIM-3 expression [48]. Tregs suppress CD8^+^ T cell secretion of IFN-γ, which would otherwise block the activation of sterol regulatory element-binding protein 1 (SREBP1)-mediated fatty acid synthesis in immunosuppressive (M2-like) tumour-associated macrophages (TAMs) [49]. ③Tregs affect effector cell function by interfering with cell metabolism mainly in the following three ways: (1) Deprivation of IL-2 in the TME, and the proliferation of Tregs and effector T cells require the maintenance of IL-2 levels. Tregs compete with effector T cells and consume a large amount of IL-2, resulting in the deficiency of IL-2 in the TME, thus inhibiting the growth of effector T cells [26, 50]. (2) CD39 and CD73 are nucleases constitutionally expressed in human and mouse Tregs. They can hydrolyse extracellular ATP or ADP into AMP and produce adenosine. Adenosine is a known inhibitory molecule that transmits inhibitory signals through different adenosine receptors (A1, A2A, A2B, and A3). Tregs promote the production of adenosine in the TME by producing the extracellular enzymes CD39 and CD73 and induce inhibitory and anti-proliferative effects by binding to the adenosine receptor A2A on the surface of effector cells [51] (Fig. 2). In addition, IL-27 signalling in Tregs critically contributes to the tumorigenic properties of Tregs via the upregulation of CD39 [52, 53]. (3) Tregs transfer a large number of cAMP to effector T cells through gap junctions to interfere with their metabolism. However, whether tumour Tregs function through this pathway remains to be determined through further study. ④The differentiation and proliferation of Tregs has a regulatory impact on DCs. Treg-expressing CTLA-4 combines with CD80 and CD86 on the surface of DCs to downregulate synergistic stimulus signalling. Lymphocyte activation gene 3 (LAG3) molecules expressed by Tregs can inhibit the expression of MHC II molecules in DCs. DC tolerance can be induced by either of these two methods, with the latter further inhibiting T cell capacity through IDO. In addition, Tregs induce CD4^−^ NKT cell anergy and suppress NKT cell cytotoxic functions through cell-to-cell contact and are mediated via impaired DC maturation [54, 55]. ⑤Factors produced by both MDSCs and Tregs form positive feedback loops to facilitate the expansion of each population and reinforce the suppressive environment. On the one hand, MDSCs induced by tumour progression selectively promoted the proliferation of Tregs in a TGF-β-dependent manner in vivo [56], and also promote the production of Tregs via CD73 highly expressed in MDSCs, which can produce adenosine by hydrolysis and further binds to A2aR, and augment the immunosuppressive effects [51, 57]. MDSCs induce the development of Tregs in vitro and in tumour-bearing mice and that Tregs induction was dependent on MDSC-secreted IL-10 and IFN-γ [58, 59]. In TME, the high expression of IDO can lead to the decrease of tryptophan and the accumulation of kynurenine, inhibit the activation of T cells and induce the production of Tregs [60, 61]. In chronic lymphocytic leukaemia (CLL), monocytic MDSCs express high levels of IDO, resulting in a decrease in T-cell proliferation and an increase in Tregs induction [62]. In a mouse colon carcinoma model, IFN-γ activated Gr-1^+^ CD115^+^ M-MDSC were shown to up-regulate MHC-II and produce IL-10 and TGF-β to mediate the development of Tregs [58]. In addition, MDSCs affects Tregs through cell-surface molecular interactions to promote the induction of Tregs, including CD40/CD40L, PD-1/PD-L1, and CD80/CTLA-4. Expression of CD40 on MDSCs is required to induce T-cell tolerance and Tregs accumulation [63, 64]. In mouse ovarian cancer model, MDSC enhanced the expression of CD80 through direct contact with tumour cells, and CD80 could bind to CTLA-4 on Tregs to enhance the immunosuppressive function of Tregs [65]. On the other hand, Tregs can also modulate MDSCs expansion and function. Tregs potentiated both the expansion of MDSCs and suppressive functions through a TGF-β-dependent mechanism [66]. IL-35 is a heterodimer of EBV-induced gene 3 (EBI3) and of the p35 subunit of IL-12, and has been identified as an inhibitory cytokine produced by natural Tregs. IL-35-producing Tregs promote the immunosuppressive capacity of MDSCs via the PD-L1 pathway [67]. Interestingly, the combination signal transduced via PD-L1 and CD169 is indispensable for the induction of IL-35^+^ Tregs [68, 69] (Fig. 2).

**Fig. 3:**
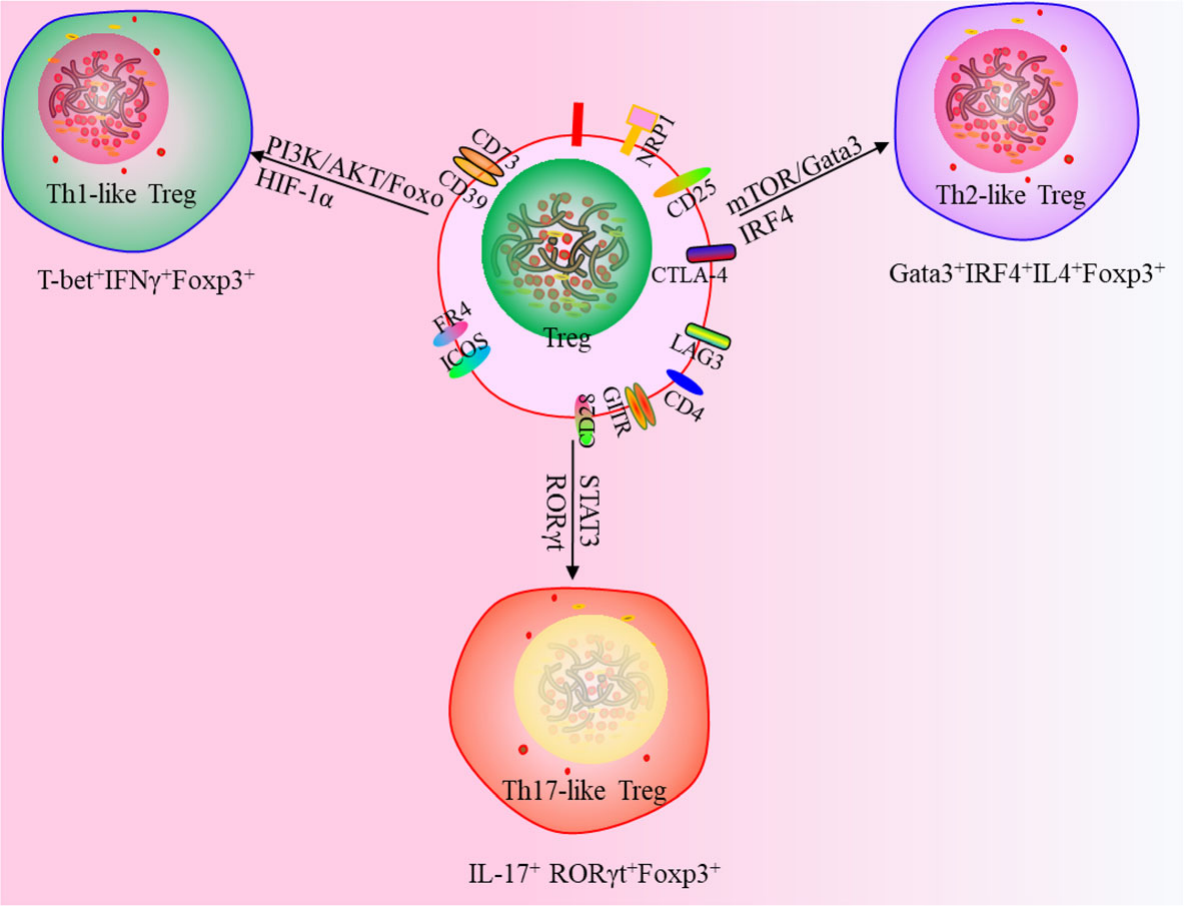
New classifications of Tregs. Th-like Tregs are Th1-like Tregs (T-bet+IFNγ+Foxp3+), Th2-like Tregs (Gata3 + IRF4 + IL4 + Foxp3+) and Th17-like Tregs (IL-17+ RORγt+Foxp3+)

A study defined Tregs using a new strategy in which Th-like Tregs subsets were characterized to further delineate their biological function and tissue distribution with a focus on their possible contribution to disease states; for example, Th1-like Tregs (T-bet^+^IFNγ^+^Foxp3^+^), Th2-like Tregs (Gata3^+^IRF4^+^IL4^+^Foxp3^+^) and Th17-like Tregs (IL-17^+^ RORγt^+^Foxp3^+^) (Fig. 3) provide new ideas for targeted Tregs therapy. Th1-like Tregs express T-bet and CXCR3. In fact, increased expression of IFN-γ by Tregs can markedly enhance checkpoint blockade therapy [70, 71]. Th2-like Tregs are characterized by the expression of Gata3 and IRF4, as well as the production of IL-4 and IL-13. Th2-like programming can be induced by IL-4R signalling that promotes Gata3 expression [72, 73]. RNA-seq and functional assays revealed that Th2-like Tregs display greater viability and enhanced autocrine IL-2-mediated activation than other subsets. Th2-like Tregs are preferentially found in tissues, rather than in circulation, and exhibit the highest migratory capacity towards chemokines enriched in tumours, which may play a role in maintaining a tumorigenic environment. Compared to healthy tissue, Th2-like Tregs are specifically enriched in malignant tissues from patients with melanoma and colorectal cancer [74]. Th17-like Tregs co-express RORγt with Foxp3 and can be generated in the periphery from conventional T cells. These IL-17-producing Treg cells retain suppressive function [75–77].

## New function and regulatory mechanisms for Tregs

### New metabolic mechanisms

**Fig. 4:**
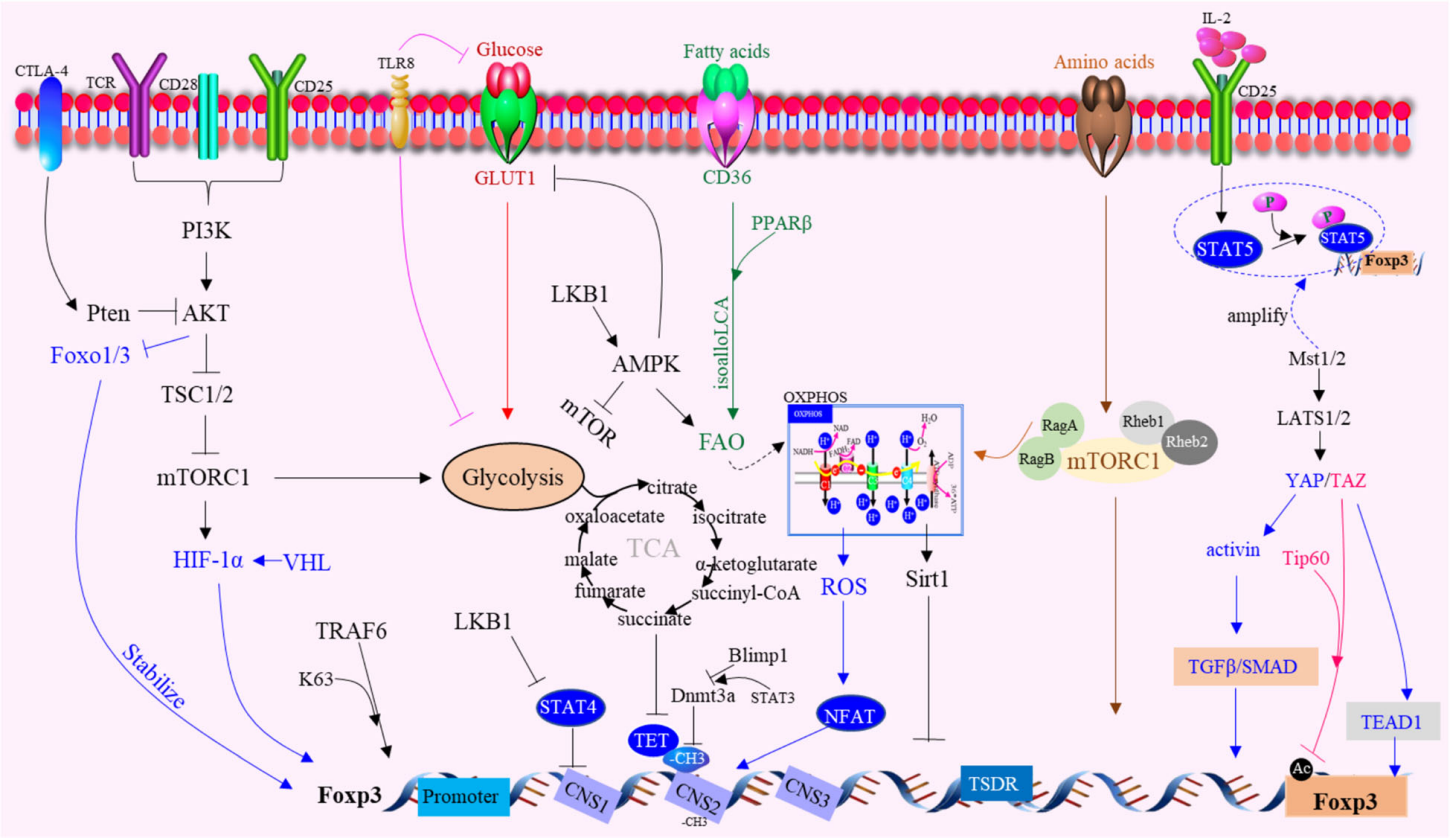
Metabolic regulation of Foxp3 expression. Environmental metabolites, intracellular metabolic intermediates and signaling pathways all regulate Foxp3 expression in Tregs. LKB1 prevents STAT4 activation and binding to CNS2 of Foxp3 gene, thus preventing the destabilization effect. E3 ubiquitin ligase VHL can regulate HIF-1α to maintain the stability and suppressive capacity of Tregs. Foxp3 opposed PI3K-Akt-mTORC1 signaling to decrease glycolysis and anabolic metabolism while increasing oxidative and catabolic metabolism. CD36 finetuned mitochondrial fitness via peroxisome proliferator-activated receptor-beta (PPARβ) signaling, programming Tregs to adapt to a lactic acidenriched TME. The deletion of TRAF6 in Tregs were resistant to implanted tumors and displayed enhanced antitumor immunity due to that Foxp3 undergoes K63-linked ubiquitination at lysine 262 mediated by the E3 ligase TRAF6. The specific ablation of RagA-RagB or Rheb1Rheb2 in Tregs has reduced Tregs accumulation and function. RagA-RagB regulated mitochondrial and lysosomal fitness, while Rheb1Rheb2 enforced Tregs suppressive gene signature licensed by amino acids. YAP-dependent upregulate activin signaling, which amplifies TGFβ/SMAD activation in Tregs. TAZ attenuated Tregs development by decreasing acetylation of Foxp3 mediated by the histone acetyltransferase Tip60. TEAD1 expression and sequestration of TAZ from the transcription factors Foxp3 promotes Tregs differentiation. TLR8 signaling selectively inhibits glucose uptake and glycolysis in human Tregs, resulting in reversal of Tregs suppression. Mst1promote Tregs migration and access to IL-2 and activity of the small GTPase Rac, which mediated downstream STAT5 activation. Mst1-Mst2 sensed IL-2 signals to promote the STAT5 activation necessary for Tregs homeostasis and lineage stability and to maintain the highly suppressive pSTAT5+Tregs

Metabolic programmes orchestrate Tregs stability, function and differentiation (Fig. 4). Central to Tregs activation are changes in lipid metabolism that support their survival and function, which can be facilitated by fatty acid-binding proteins (FABPs), a family of lipid chaperones required for the uptake and intracellular trafficking of lipids [78]. The administration of isoalloLCA from bile acid (BA) metabolites to mice increased Tregs differentiation [79]. Furthermore, both dietary and microbial factors influenced the composition of the gut BA pool and modulated an important population of colonic Foxp3^+^ Tregs expressing the transcription factor RORγ [80, 81]. Although Tregs in the tumour milieu rely on supplemental energy routes involving lipid metabolism, another study showed that both glycolytic and oxidative metabolism contributed to Tregs expansion because of the relative advantage of intratumoural Tregs in glucose uptake that may fuel FA synthesis [82]. Tregs can also undergo apoptosis, and such apoptotic Tregs release and convert large amounts of ATP to adenosine via CD39 and CD73 and mediate immunosuppression through the adenosine-A2A pathways [44, 83]. Notably, Tregs in visceral adipose tissue (VAT) show pronounced sexual dimorphism. Male VAT facilitates the recruitment of Tregs via the CCL2/CCR2 axis. Sex hormones also regulate VAT inflammation, which shape the transcriptional landscape of VAT-resident Tregs in a Blimp1 transcription factor-dependent manner [84]. Tregs exhibit a unique metabolic profile characterized by an increase in mitochondrial metabolism relative to other CD4^+^ T effector subsets. Mitochondrial transcription factor A (Tfam) is essential for mitochondrial respiration and maintains Tregs functions by controlling mitochondrial DNA replication, transcription, and packaging in tumours. Furthermore, Foxp3 has also been shown to promote respiration [85]. The specific ablation of mitochondrial respiratory chain complex III in Tregs results in the loss of T cell-suppression capacity without altering Tregs proliferation and survival and leads to decreased expression of genes associated with Tregs function with Foxp3 expression remaining stable. Loss of complex III in Tregs increases the methylation of DNA as well as the metabolites 2-hydroxyglutarate (2-HG) and succinate, which inhibit the ten-eleven translocation (TET) family of DNA demethylases. Therefore, mitochondrial complex III is required for maintaining the expression of immune regulatory genes Tregs suppressive functions [86]. It is noteworthy that the regulatory function can be re-established in Foxp3-deficient Tregs by targeting their metabolic pathways [87]. Foxp3 deficiency leads to the dysregulation of metabolic checkpoint kinase mammalian target of rapamycin (mTOR) complex 2 (mTORC2) signalling and enhanced aerobic glycolysis and oxidative phosphorylation. The specific deletion of the mTORC2 adaptor gene Rictor in Foxp3-deficient Tregs showed increased viability in a Foxo1 transcription factor-dependent manner, re-establishing a subset of Tregs genetic circuits and suppressing the Teff cell-like glycolytic and respiratory programmes and thus contributing to immune dysregulation. Treatment of Foxp3-deficient Tregs with mTOR inhibitors similarly antagonized their Teff cell-like programme and restored their suppressive function [88]. It is noteworthy that CD36 was selectively upregulated in intratumoural Tregs, serving as a central metabolic modulator. CD36 fine-tuned mitochondrial fitness via peroxisome proliferator-activated receptor-beta (PPARβ) signalling, programming Tregs to adapt to a lactic acid-enriched TME. Genetically induced and specific deletion of *Cd36* in Tregs suppressed tumour growth, decreased in intratumoural Tregs levels and enhanced the antitumour activity of TILs without disrupting immune homeostasis. Moreover, CD36 targeting elicited additive antitumour responses during anti-PD-1 therapy [89]. Liver kinase B1 (LKB1) programmes the metabolic and functional fitness of Tregs in the control of immune tolerance and homeostasis and functions as a critical inhibitor of DCs immunogenicity, and when lost, mitochondrial fitness is reduced and maturation, migration, and T cell priming of peripheral DCs are increased. Loss of LKB1 specifically primes thymic CD11b^+^ DCs to facilitate thymic Tregs development and expansion, which is independent from AMPK signalling but dependent on mTOR and enhanced phospholipase C β1-driven CD86 expression [90, 91]. The specific deletion of LKB1 in Tregs can induce a fatal inflammatory disease characterized by excessive Th2-type-dominant responses, which not only disrupts the survival, mitochondrial fitness and metabolism of Tregs but also induces aberrant expression of immune regulatory molecules, including PD-1 and the TNF receptor superfamily proteins GITR and OX40. Unexpectedly, the LKB1 function in Tregs was found to be independent of conventional AMPK signalling and the mTORC1-HIF-1α axis but contributed to the activation of β-catenin signalling for the control of PD-1 and TNF receptor proteins [92]. Deficiency of KAP1, a binding partner of Foxp3, in Tregs led to failure to induce Foxp3-regulated Tregs signature genes because of the decreased expression of Slc1a5, whose reduced expression resulted in reduced mTORC1 activation [93]. Mechanistically, mTOR functions downstream of antigenic signals to drive IRF4 expression and mitochondrial metabolism, and accordingly, deletion of Tfam severely impaired Tregs suppressive function and eTregs generation [94]. Lysosomal TRAF3IP3 acts as a pivotal regulator in the maintenance of Tregs metabolic fitness. Treg-specific deletion of Traf3ip3 impairs Tregs function, causing stronger antitumour T cell responses in mice. TRAF3IP3 restricts mTORC1 signalling by recruiting the serine-threonine phosphatase catalytic subunit (PP2Ac) to the lysosome, thereby facilitating the interaction of PP2Ac with the mTORC1 component Raptor [95, 96]. mTOR activity has been observed to be increased in Tregs, and the genetic deletion of Raptor inhibits Tregs function. The inhibition of mTOR during T cell activation promotes the generation of long-lived central Tregs with a memory-like phenotype in mice. Metabolically, these central memory Tregs possess enhanced spare respiratory capacity, similar to CD8^+^ memory cells. Indeed, the genetic deletion of Rptor leads to decreased expression of ICOS and PD-1 on eTregs [97]. TLR signals that promote Tregs proliferation increase PI(3)K-Akt-mTORC1 signalling, glycolysis and Glut1 expression. However, TLR-induced mTORC1 signalling also impairs Tregs suppressive capacity. In contrast, Foxp3 counters PI(3)K-Akt-mTORC1 signalling to decrease glycolysis and anabolic metabolism while increasing oxidative and catabolic metabolism. Notably, Glut1 expression is sufficient to increase the number of Tregs, but it reduces their suppressive capacity and Foxp3 expression [98]. Furthermore, TLR8 signalling selectively inhibits glucose uptake and glycolysis in human Tregs, resulting in the reversal of Tregs suppression. Importantly, TLR8 signalling-mediated reprogramming of glucose metabolism and function in human Tregs can enhance antitumour immunity in vivo in a melanoma adoptive transfer T cell therapy model [99]. Amino acids can license Tregs function by priming and sustaining TCR-induced mTORC1 activity. mTORC1 activation can be induced by amino acids, especially arginine and leucine. Rag and Rheb GTPases are central regulators of amino acid-dependent mTORC1 activation in effector Treg (eTregs) cells. Mice bearing the specific ablation of RagA-RagB or Rheb1Rheb2 in Tregs had reduced eTregs accumulation and function. RagA-RagB regulated mitochondrial and lysosomal fitness, while Rheb1Rheb2 enforced the eTregs suppressive gene signature [100]. Notably, Tregs in peripheral tissues, including tumours, are more sensitive to Rag GTPase-dependent nutrient sensing. Ablation of RagA alone impairs Tregs accumulation in tumours, resulting in enhanced antitumour immunity [101].

### New genetic mechanisms

The preferential differentiation of human foetal CD4^+^ naïve T cells to Tregs can be enhanced by Helios [102], as a key transcription factor that stabilizes Tregs in during inflammatory responses, providing a genetic explanation for a core property of Tregs [103]. Helios-deficient Tregs within tumours acquire effector T cell function and contribute to the immune responses against cancer by upregulating effector cytokines, which show high affinity for self-antigens, as detected by both increased GITR/PD-1 expression and increased responsiveness to self-antigens [104]. In both Tregs and conventional T cells (Tconv cells), Foxp1, a forkhead transcription factor and a sibling of Foxp3, occupies a large number of Foxp3-bound genomic sites. The absence of Foxp1 in Tregs results in impaired function and competitive fitness and has been associated with significantly decreased CD25 expression and IL-2 responsiveness, decreased CTLA-4 expression, and increased SATB1 expression [105]. Selective demethylation of the Treg-specific demethylated region (TSDR) in the Foxp3 gene can stabilize Foxp3 expression and is a defining characteristic of nTregs [106]. Conserved noncoding sequence 2 (CNS2), a dedicated Foxp3 intronic element, can maintain Tregs lineage identity by acting as a sensor of IL-2 and its downstream target STAT5, thereby Foxp3 expression is sustained during maturation [107]. CNS2 can also be heavily methylated when Blimp1 is ablated, leading to a loss of Foxp3 expression. Blimp1 negatively regulates IL-6- and STAT3-dependent Dnmt3a expression and function, restraining the methylation of CNS2 at the Foxp3 locus [108]. CNS3, another intronic Foxp3 enhancer, acts as an epigenetic switch, poising the Foxp3 promoter in precursor cells to commit the Tregs to be responsive to TCR stimuli. CNS3-dependent expansion of the TCR repertoire enables Tregs to control self-reactive T cells effectively, especially when thymic negative selection is genetically impaired [109]. YAP, a coactivator of the Hippo pathway, is highly expressed in Tregs and boosts Foxp3 expression and function. This potentiation is based on YAP-dependent upregulation of activin signalling, which amplifies TGFβ/SMAD activation in Tregs. YAP deficiency resulted in dysfunctional Tregs unable to suppress antitumour immunity or promote tumour growth in mice [110]. TAZ, a coactivator of TEAD transcription factors inducing Hippo signalling attenuated Tregs development by decreasing the acetylation of Foxp3 mediated by the histone acetyltransferase Tip60, which targeted Foxp3 for proteasomal degradation. In contrast, under Treg-skewing conditions, TEAD1 expression and the sequestration of TAZ from the transcription factors RORγt and Foxp3 promoted Tregs differentiation [111]. Nuclear receptor Nr4a, a key transcription factor maintaining Tregs genetic programmes, contributes to Treg-mediated suppression of antitumour immunity in the TME. The specific ablation of Nr4a1 and Nr4a2 in Tregs conferred resistance to tumour growth, and triggered the effector activities of CD8^+^ CTLs [112]. CBP/p300 are closely related acetyltransferases and transcriptional coactivators. CBP/p300 acetylates prostacyclin synthase, which regulates Tregs differentiation by altering pro-inflammatory cytokine secretion by T and B cells in follicular lymphoma [113]. The pro-autophagy protein AMBRA1 is also a key modulator of T cells, regulating the complex network that leads to human Tregs differentiation and maintenance. Indeed, AMBRA1 promotes the stability of the transcriptional activator FOXO3, which, in turn, triggers Foxp3 transcription through its ability to interact with the phosphatase PP2A [114]. Disruption of H3K27 methyltransferase EZH2 activity in Tregs, drove the pro-inflammatory functions of TI-Tregs, remodelling the TME and enhancing the recruitment and function of CD8^+^ and CD4^+^ effector T cells that eliminate tumours [115]. SUMO-specific protease 3 (SENP3) is a pivotal regulator of Tregs that functions by controlling the SUMOylation and nuclear localization of BACH2. Treg-specific deletion of Senp3 results in T cell activation and enhanced antitumour T cell responses. SENP3-mediated BACH2 deSUMOylation prevents the nuclear export of BACH2, thereby suppressing the genes associated with CD4^+^ T effector cell differentiation and stabilizing Treg-specific gene signatures [116].

### New molecular mechanisms

Some new advances in the molecular mechanisms of Tregs have been achieved (Fig. 1). Tregs, which have abundant expression of the IL-2 receptor (IL-2R), are dependent on IL-2 produced by activated T cells, indicating that the consumption of IL-2 by Tregs is related to their suppressor function. The capture of IL-2 was dispensable for the control of CD4^+^ T cells and that IL-2R-dependent activation of the transcription factor STAT5 had an essential role in the suppressor function of Tregs [117]. IL-2 and its downstream transcription factor STAT5 are important for maintaining the homeostasis and function of Tregs. The serine-threonine kinase Mst1 has been identified as a signal-dependent amplifier of IL-2-STAT5 activity in Tregs. High Mst1 and Mst2 (Mst1-Mst2) activity in Tregs is crucial to prevent tumour resistance and autoimmunity. Mst1 deficiency limited Tregs migration and access to IL-2 and the activity of the small GTPase Rac, which mediated downstream STAT5 activation. Mst1-Mst2 sensed IL-2 signals to promote the STAT5 activation necessary for Tregs homeostasis and lineage stability and to maintain the highly suppressive pSTAT5^+^ Tregs [118]. During T cell activation, phosphorylation of Foxp3 in Tregs can be regulated by a TAK1-Nemo-like kinase (NLK) signalling pathway. When Foxp3 is phosphorylated, Tregs are stabilized because Foxp3 cannot associate with the Stub1 E3-ubiquitin protein ligase [119]. Additionally, the specific deletion of the Tregs kinase TAK1 can decrease the number of Tregs in the peripheral lymphoid organs. Furthermore, TAK1 is crucial for the survival of Tregs [120]. E3 ubiquitination ligase TRAF6-deficient Tregs were dysfunctional in vivo. Mice with restricted deletion of TRAF6 in Tregs were resistant to implanted tumours and displayed enhanced antitumour immunity because Foxp3 underwent K63-linked ubiquitination at lysine 262 as mediated by TRAF6 [121]. Suppression of tumorigenicity 2 (ST2) is regarded as the only receptor of IL-33. Infiltrated ST2-expressing Tregs were responsive to IL-33, and the percentage of Tregs was increased upon IL-33 stimulation, in particular Foxp3^+^GATA3^+^ Tregs, which enhanced the suppressive functions of Tregs by inducing IL-10 and TGF-β1 and decreasing the proliferation of responder T cells in head and neck squamous cell carcinoma [122, 123]. Another scRNA-seq longitudinal profile indicated that interferon-responsive Tregs were more prevalent early in tumour development, whereas a specialized effector phenotype characterized by enhanced expression of ST2 was predominant in advanced disease. The specific deletion of ST2 in Tregs alters the evolution of effector Tregs diversity, increases the infiltration of CD8^+^ T cells into tumours, and decreases tumour burden [124, 125]. In mice with colorectal cancer (CRC), tumour-infiltrating Tregs preferentially upregulated ST2, and IL-33/ST2 signalling positively correlated with tumour burden, which favours their accumulation in the TME and concomitantly restrains the frequencies of effector CD8^+^ T cells [126]. Activated Tregs express the surface receptor glycoprotein-A repetitions predominant (GARP), which binds and activates latent TGF-β. GARP^−/−^ Tregs were significantly reduced in the gut and exhibited a reduction in CD103 expression, a colon-specific migratory marker [127, 128]. Integrins, consisting of α and β subunits that mediate cell-to-cell and cell-to-extracellular matrix interactions, play crucial roles in facilitating Tregs contact-mediated suppression. Activation of integrin α4β1 can increase the suppressive capacity of Tregs [129, 130]. A RNA-seq analysis of human Tregs revealed β-catenin as a key regulator of IFN-γ and IL-10 expression. The activated β-catenin signature was enriched in human IFN-γ^+^ Tregs, as confirmed in vivo with Treg-specific β-catenin-stabilized mice [131]. Significantly, Tregs homeostasis is critically linked to mucosa-associated lymphoid tissue 1 (Malt1) function via Tregs intrinsic and extrinsic mechanisms. TCR-mediated Malt1 proteolytic activity and self-cleavage were found to drive IL-2 expression in conventional CD4^+^ T cells, thereby regulating the amount of IL-2 available for Tregs maintenance of homeostasis [132]. In contrast, CARD11-BCL10-MALT1 (CBM) signalling is essential for mediating the suppressive function of Tregs in a Malt1 protease-dependent manner. In malignant melanoma models, the acute selective genetic blockade of BCL10 signalling in Tregs or pharmacological Malt1 inhibition enhanced antitumour immune responses [133]. Upon disruption of CBM, the majority of TI-Tregs produce IFN-γ, followed by stunted tumour growth [70]. Treg-specific deletion of Bcl11b showed decreased functional marker levels under homeostatic conditions, during inflammation, and in tumours [134]. Additionally, genome-wide occupancy studies coupled with gene expression profiling revealed that Bcl11b, in association with Foxp3, is critical for establishing a Treg-specific gene activation programme. Furthermore, Bcl11b restricts misdirected recruitment of Foxp3 to sites, which would otherwise cause an altered transcriptome profile of the Tregs [135].

## Tumour immunotherapy strategies targeting Tregs

### Depletion of Tregs

Foxp3^+^ Tregs suppress antitumour responses in endogenous lymphoma. Ablation of Foxp3^+^ Tregs significantly delayed tumour development. The ratio of Tregs to effector T cells was elevated in growing tumours [136]. The infiltration of T cells into the TME represents a critical bottleneck for immune-mediated control of cancer. This bottleneck can be overcome by depleting immunosuppressive Foxp3^+^ Tregs, which can lead to an increased frequencies of TILs by promoting the development of high endothelial venules (HEVs). Tregs depletion results not only in widespread disruption to HEV networks in LNs but also in CD8^+^ T cell activation, which subsequently drives intratumoural HEV development. Tregs depletion enables a self-amplifying loop of T-cell activation, which promotes HEV development, T-cell infiltration and, ultimately, tumour destruction [137]. Tregs depletion leads to increased production of the CXCR3 ligand CXCL10 from endothelial cells in tumours. Furthermore, T cells migrate into intestinal tumours through CXCR3. Tregs reduce endothelial CXCL10 production and inhibit T-cell migration into tumours, and CXCR3-mediated signalling is crucial for lymphocyte accumulation in intestinal tumours. Therefore, immunotherapy aimed at Tregs depletion may be effective by increasing not only effector T cell activity but also their accumulation in tumours [138]. Depletion of Tregs in enterotoxigenic *Bacteroides fragilis* (ETBF)-colonized mice enhanced colitis but diminished the tumorigenesis associated with the shifting of the mucosal cytokine profile from IL-17 to IFN-γ. However, blocking IL-2 restored Th17 responses and tumour formation after Tregs depletion, indicating that Tregs restrain the availability of IL-2 in the local microenvironment, allowing the development of the Th17 cells necessary to promote ETBF-triggered neoplasia [139]. Tregs are abundant in pancreatic cancer. Tregs depletion fails to relieve immunosuppression and leads to accelerated tumour progression. Tregs are a key source of TGF-β ligands, and accordingly, their depletion reprogrammed the fibroblast population, with loss of tumour-restraining, smooth muscle actin-expressing fibroblasts. In contrast, an increase in the chemokines CCL3, CCL6, and CCL8 led to the recruitment of myeloid cells, restoration of immune suppression, and promotion of carcinogenesis [140]. An immunotoxin (2E4-PE38) that kills mouse cells expressing CD25 by attaching the Fv portion of monoclonal antibody 2E4 (anti-mouse CD25) to a 38-kDa portion of Pseudomonas exotoxin A has been produced. The number of Tregs were significantly reduced in the 2E4-PE38-injected tumours but not in the spleen. Injected tumours showed an increase in CD8^+^ T cells expressing IFN-γ, the activation markers CD69 and CD25, and macrophages and conventional dendritic cells. Selective depletion of Tregs in tumours facilitates the development of a CD8^+^ T cell-dependent antitumour effect [141]. Anti-VEGF therapy prolongs recurrence-free survival in patients with glioblastoma but does not improve overall survival. More Tregs were observed in the spleens of tumour-bearing mice and later in tumours after anti-VEGF treatment. Elimination of Tregs through a CD25 blockade administered before anti-VEGF treatment restored IFN-γ production in the CD8^+^ T cells and improved the antitumour response from anti-VEGF therapy [142]. Although IL-2 is important for effector T cell function, it has been hypothesized that therapies blocking IL-2 signals weaken Tregs activity, promoting immune responses using anti–IL-2 or anti–IL-2R Abs. Treatment with an IL-2 mutein reduces Tregs numbers and impairs tumour growth in mice [50]. Patients over 60 years old responded more efficiently to anti-PD-1, and the likelihood of generating a response to anti-PD-1 treatment increased with age. Compared with the older mice, the young mice with the same tumours had a significantly higher population of Tregs. Depletion of Tregs using anti-CD25 increased the response to anti-PD-1 in the young mice [143]. CD4^+^ T cell transfer into lymphodepleted animals or Tregs depletion promoted GzmB expression by tumour-infiltrating CD4^+^, an effect prevented by IL-2 neutralization. Transcriptional analysis revealed a polyfunctional helper and cytotoxic phenotype characterized by the expression of T-bet and Blimp-1. While T-bet ablation restricted the production of IFN-γ, loss of Blimp-1 prevented GzmB expression in response to IL-2, suggesting that two independent programmes required for the polyfunctionality of tumour-reactive cytotoxic CD4^+^ T cells [144]. In addition, blocking IFN α and β receptor 1 (IFNAR1) on Tregs significantly decreases both the Tregs immunosuppressive function and myeloma progression. Selective depletion of Tregs led to the complete remission and prolonged survival of mice injected with myeloma cells [145]. Additionally, localized Tregs depletion led to a significant reduction in lung tumours. The immune response after Tregs depletion in tumours showed the restoration of NK cell activity, enhanced Th1 activity, and an increase in CD8^+^ cytotoxic T cell response [146]. Imatinib, a tyrosine kinase inhibitor of the oncogenic BCR-ABL protein expressed by chronic myelogenous leukaemia (CML) cells, shows off-target effects, including on Lck expressed in T cells. Imatinib-treated CML patients in complete molecular remission (CMR) exhibited selective depletion of effector Tregs (eTregs) and a significant increase in effector/memory CD8^+^ T cells, while non-CMR patients did not show these effects. Mechanistically, because of the much lower expression of Foxp3-dependent Lck and ZAP-70 in Tregs compared with other T cells, imatinib inhibition of Lck also reduced their TCR signal intensity, rendering them selectively susceptible to signal-deprived apoptosis [147]. Zoledronic acid (ZA) treatment resulted in a selective decrease in the frequency of Tregs that was associated with a significant increase in the proliferation of T cells and NKs in the peripheral blood of patients with metastatic cancer. Furthermore, the colocalization of nuclear factor of activated T cells (NFAT) and Foxp3 was significantly reduced in Tregs upon ZA treatment [148]. CD25 expression is largely restricted to TI-Tregs in mice and humans. While anti-CD25 mAbs were observed to deplete Tregs in the periphery, the upregulation of inhibitory Fc gamma receptor (FcγR) IIb at the tumour site prevented TI-Tregs depletion, which may underlie the lack of antitumour activity. Use of an anti-CD25 antibody with enhanced binding to activate FcγRs led to effective depletion of TI-Tregs, increased effector/Tregs ratios, and improved control of established tumours [149]. Additionally, a novel determinant of antitumour activity using fusion proteins consisting of IL-2 and an antibody Fc region (IL-2-Fc fusion proteins) effectively prevented unwanted the activation of CD25^+^ Tregs and resulted in the profound expansion of CD25 cytotoxic subsets. The efficacy was crucially dependent on the depletion of Tregs through Fc-mediated immune effector functions [150].

Blocking the migration of Tregs to the TME is another promising strategy for tumour immunotherapy because it also reduces the infiltration of Tregs. The induction of oncogenic BRAF^V600E^ and loss of Pten in melanocytes led to localized accumulation of Foxp3^+^ Tregs but not CD8^+^ T cells. In melanoma, CCR4 was required for the homing of Tregs to nascent tumour sites from LNs. BRAF^V600E^ signalling in melanocytes controlled the expression of CCR4-cognate chemokines and governed the recruitment of Tregs to tumour-induced skin sites. Tregs depletion enhances immunosurveillance, as shown by CD8^+^ T cell responses against the tumour/self-antigen gp100, induced concurrently with the formation of microscopic neoplasia [151]. Mogamulizumab, a de-fucosylated anti-CCR4 antibody, reduces the levels of CCR4^+^ T cells and CCR4^+^ Tregs in patients with cutaneous T-cell lymphoma (CTCL), which may in turn improve their immune profiles [152]. Tregs accumulation in the leukaemic haematopoietic microenvironment (LHME) has adverse impacts on patient outcomes. Both local expansion and migration accounted for Tregs accumulation in the LHME. Moreover, blocking the CCL3-CCR1/CCR5 and CXCL12-CXCR4 axes inhibited Tregs accumulation in the LHME and delayed leukaemia progression [153]. CRC cell-secreted CCL20 can recruit Tregs to promote chemoresistance via FOXO1/CEBPB/NF-κB signalling, indicating that the FOXO1/CEBPB/NF-κB/CCL20 axis might provide a promising target for CRC treatment. Thus, CCL20 will be a potential target [154]. Notably, compared to Tregs from healthy tissues, TI-Tregs more substantially downregulated Foxo1 target genes. A relatively low level of Foxo1-mutant expression was sufficient to deplete TI-Tregs, activate effector CD8^+^ T cells, and inhibit tumour growth without inducing autoimmunity. Thus, Foxo1 inactivation is essential for the migration of aTregs [155]. Cancer-Foxp3 was positively correlated with Tregs accumulation in tumour tissues derived from PDAC patients and was associated with tumour volume and prognosis. CCL5 was directly transactivated by cancer-Foxp3 and promoted the recruitment of Tregs from peripheral blood to the tumour site. Tregs recruitment by cancer-Foxp3 was impaired by the neutralization of CCL5, inhibiting the growth of PDAC [156]. Human Tregs express CCR4 and can be recruited to the TME through CCL17 and CCL22. In some cancers, Tregs accumulation correlates with poor patient prognosis [157]. Infiltration of Tregs was caused by the interaction between the tumour-producing chemokine CCL17 and receptor CCR4 expressed on Tregs in dogs bearing spontaneous bladder cancer. CCR4 blockade inhibited tumour growth and Tregs infiltration into tissues with improved survival and a low incidence of clinically relevant toxicity [158]. Additionally, intratumoural IFN-α gene delivery reduced the trafficking of Tregs to the tumour through the downregulation of tumour CCL17 expression [159]. CCL22 expression by DCs promotes the formation of cell–cell contacts and interaction with Tregs through receptor CCR4. CCL22 deficiency led to their prolonged survival upon vaccination [160]. Besides, intratumoural CCL22 is induced in tumour-infiltrating DCs through cancer cell-derived IL-1α. The IL-1 receptor antagonist anakinra or IL-1 siRNA transfect into tumour cell lines can lead to suppression of Tregs migration in pancreatic cancer and HCC [161]. Notably, in breast cancer, CCR8 were upregulated in tumour-resident Tregs compared to their levels in normal tissue-resident Tregs. Targeting CCR8 to inhibit the migration of tumour-resident Tregs might represent a promising immunotherapeutic approach for the treatment of breast cancer [162].

### Targeting immune checkpoint (IC) on Tregs

The TME confers a suppressive function on Tregs by upregulating IC molecule expression. Targeting IC molecules including CTLA-4, TIGIT, PD-1, GITR, etc. on Tregs may be effective for cancer treatment [163]. CTLA-4 was the first identified as an inhibitory immune checkpoint on Tregs and activated CD8 and CD4 effector cells. Upon high affinity binding to its ligand CD80 and CD86 on APCs, CTLA-4 limits the further activation of effector cells and plays an essential role in maintaining the suppressive function of Tregs. Therefore, the anti-CTLA-4 antibodies can remove the suppressive function of Tregs and release the cytotoxicity function of effector cells. However, compared with anti-PD therapy, CTLA-4 targeting faces two related challenges: suboptimal efficacy and increased toxicity [164]. Two mAbs, ipilimumab (IgG1) and tremelimumab (IgG2), which block the function of CTLA-4, have demonstrated durable clinical activity in a subset of patients with advanced solid malignancies by augmenting effector T-cell–mediated immune responses. Studies in mice suggest that anti-CTLA-4 mAbs may also selectively deplete intratumoral Foxp3^+^ Tregs via an Fc-dependent mechanism. Both ipilimumab and tremelimumab increase infiltration of intratumoral CD4^+^ and CD8^+^ cells without significantly changing or depleting Foxp3^+^ cells within the TME. Anti-CTLA-4 immunotherapy does not deplete Foxp3^+^ cells in human tumours, which suggests that their efficacy could be enhanced by modifying the Fc portions of the mAbs to enhance Fc-mediated depletion of intratumoral Tregs [165]. Antibodies with isotypes equivalent to ipilimumab and tremelimumab mediate intratumoral Tregs depletion in vivo, increasing the CD8/Tregs ratio and promoting tumour rejection. Antibodies with improved FcγR binding profiles drove superior antitumour responses and survival [166, 167]. Clinically effective anti-CTLA-4 mAb causes tumour rejection by mechanisms that are independent of checkpoint blockade but dependent on the host Fc receptor [168]. Sanseviero et al found other mechanisms about anti-CTLA-4 inefficacy. They found that anti-CTLA-4 binding to FcRs has been linked to depletion of intratumoral Tregs, and further coincided with activation and degranulation of intratumoral NK cells. Combination therapy with anti-CTLA-4 plus IL15/IL15Rα complexes enhanced tumour control compared with either monotherapy [169]. Fc-region–modified anti-CTLA-4 mAb with high antibody-dependent cell-mediated cytotoxicity (ADCC) and cellular phagocytosis (ADCP) activity selectively depleted CTLA-4^+^Foxp3^+^ Tregs and consequently expanded tumour antigen-specific CD8^+^ T cells. However, the ADCC strategy is unlikely to succeed in colorectal, liver, prostate and ovarian cancer treatments [170, 171]. Ipilimumab is the first immune checkpoint blockade (ICB) that was approved by FDA for treating metastatic melanomas [172]. Despite the prolonged survival of patients, anti-CTLA-4 antibody treatment can cause severe immunotherapy-related adverse effects (irAEs), which significantly limits its clinical benefits. Therefore, more studies turn to combine low-dose anti-CTLA-4 immunotherapy with the anti-PD-1 blockade. Even with a reduced dose of anti-CTLA-4 antibodies, the combination therapy with anti-PD-1 treatment can cause severe irAEs in many patients. Targeting CTLA-4 has shown remarkable long-term benefit and thus remains a valuable tool for cancer immunotherapy if the irAE can be brought under control [173, 174]. Zhang et al. found that while irAE-prone Ipilimumab and TremeIgG1 rapidly direct cell surface CTLA-4 for lysosomal degradation, the non-irAE-prone antibodies they generated, HL12 or HL32, dissociate from CTLA-4 after endocytosis and allow CTLA-4 recycling to cell surface by the LRBA-dependent mechanism. Disrupting CTLA-4 recycling results in robust CTLA-4 downregulation by all anti-CTLA-4 antibodies and confers toxicity to a non-irAE-prone anti-CTLA-4 mAb. Conversely, increasing the pH sensitivity of TremeIgG1 by introducing designed tyrosine-to-histidine mutations prevents antibody-triggered lysosomal CTLA-4 downregulation and dramatically attenuates irAE. Surprisingly, pH-sensitive anti-CTLA-4 antibodies are more effective in intratumour Tregs depletion and rejection of large established tumours by avoiding CTLA-4 downregulation and due to their increased bioavailability [175]. The combination of intratumoural injections of TLR1/2 ligand Pam3CSK4 plus anti-CTLA-4 mAb enhanced antitumour immune responses compared to the response induced by anti-CTLA-4 alone, and its efficacy depended on CD4 T cells, CD8 T cells, FcγR IV, and macrophages. Interestingly, the TLR1/2 ligand increased FcγR IV expression on macrophages, leading to antibody-dependent macrophage-mediated depletion of Tregs in melanoma and increasing efficacy of anti-CTLA-4 mAbs in the combination treatment [176]. In addition, targeting interferon signalling and CTLA-4 enhance the therapeutic efficacy of anti-PD-1 immunotherapy in preclinical model of HPV^+^ oral cancer [177]. The CTLA-4 x OX40 bispecific antibody ATOR-1015 induces antitumor effects through tumour-directed immune activation. By targeting CTLA-4 and OX40 simultaneously, ATOR-1015 is directed to the tumour area where it induces enhanced immune activation, and thus has the potential to be a next generation CTLA-4 targeting therapy with improved clinical efficacy and reduced toxicity. ATOR-1015 is also expected to act synergistically with anti-PD-1/PD-L1 therapy [178]. Genetic depletion of EZH2 in Tregs leads to robust antitumor immunity. Pharmacological modulating EZH2 expression in T cells can improve antitumor responses elicited by anti-CTLA-4 therapy [179]. Interestingly, systemic short chain fatty acids from gut microbial metabolites could limit antitumor effect of CTLA-4 blockade in hosts with cancer [180]. TIGIT signalling in Tregs directs their phenotype acquisition, and TIGIT primarily suppresses antitumour immunity via Tregs but not CD8^+^ T cells. Moreover, TIGIT^+^ Tregs upregulated the expression of TIM-3 in tumours, and TIM-3 and TIGIT synergized to suppress antitumour immune responses [181]. Androgen deprivation therapy (ADT) induces a complex pro-inflammatory infiltrate, which was apparent in the early post-castration period but diminished as castration resistance emerged. Combining ADT with TI-Tregs depletion using a depleting anti-CTLA-4 antibody significantly delayed the development of castration resistance and prolonged the survival of a fraction of tumour-bearing mice [182]. In advanced HCC, the frequency of checkpoint inhibitor-positive Tregs was inversely correlated with the age of the patients and corresponded to enhanced numbers of Tregs producing IL-10 and IL-35. Tregs inhibited the IFN-γ production and cytotoxicity of CD8^+^ T cells in which the activity was partially blocked by neutralizing PD-1 and PD-L1 antibodies, specifically in HCC patients [183]. PD-1 blockade is a cancer immunotherapy that has been effective in various types of cancer. In a fraction of treated patients, however, it causes rapid cancer progression called hyperprogressive disease (HPD) with the production of anti–PD-1 mAb. Tumour-infiltrating Foxp3^hi^CD45RA^−^CD4^+^ eTregs expressed PD-1 at much higher levels than circulating eTregs, which were abundant and highly suppressive in tumours. A comparison of GC tissue samples before and after anti-PD-1 mAb therapy revealed that the treatment markedly increased tumour-infiltrating proliferative eTregs in HPD patients. Functionally, circulating and tumour-infiltrating PD-1^+^ eTregs were highly activated, showing higher expression of CTLA-4 than shown by PD-1^−^ eTregs. PD-1 blockade significantly enhanced the suppressive activity of Tregs in vitro [184]. Agonistic mAbs targeting GITR exert potent therapeutic activities in preclinical tumour models. Anti-GITR mAbs are thought to act by depleting and destabilizing the TI-Tregs population. Further characterization of persisting Tregs following anti-GITR mAb treatment showed that a highly activated subpopulation of CD44^hi^ ICOS^hi^ TI-Tregs were preferentially targeted for elimination, with the remaining Tregs exhibiting a less suppressive phenotype. With these changes in Tregs, intratumoural CD8^+^ T cells acquired a more functional phenotype characterized by their ability to downregulate PD-1 and LAG-3. This reversal of CD8^+^ T-cell exhaustion was dependent on both agonistic GITR signalling and Tregs depletion [185]. An in vitro blockade of PD-1 increased Tregs percentages and pSTAT3 expression and reduced Treg-suppressive function. PD-1 blockade also led to IL-10 production by T cells, resulting in higher Tregs proliferation. The addition of a STAT3 inhibitor ameliorated the increase in Tregs, enhanced suppressive function, and decreased T-cell IL-10 production in vitro [186]. STAT3 binds through its N-terminal floppy domain to the exon 2 β sheet region of Foxp3 to form STAT3-Foxp3 complexes, extending the co-transcriptional activity of Foxp3 to other STAT3-target genes that lack Foxp3-binding sites [187, 188]. Glioblastoma promotes immunosuppression through the upregulation of PD-L1 and Tregs expansion, indicating that PD-L1 may expand and maintain immunosuppressive Tregs, which are associated with decreased survival of patients. A blockade of the PD-L1/PD-1 axis may reduce the expansion of Tregs and further improve T cell function [189]. Claudin-low breast cancer is an aggressive subtype. Despite adaptive immune cell infiltration in claudin-low tumours, treatment with anti-CTLA-4 and anti-PD-1 antibodies cannot efficiently control tumour growth. CD4^+^Foxp3^+^ Tregs represented a large proportion of TILs in claudin-low tumours, and Tregs isolated from tumours were able to suppress effector T cell responses. Tregs in the TME highly expressed PD-1 and were recruited partly through tumour-derived CXCL12. Antitumour efficacy requires stringent Tregs depletion combined with checkpoint inhibition [190]. Despite high PD-1 expression, TIM-3^+^ TI-Tregs display a greater capacity to repress the proliferation of naive T cells than TIM-3^−^ Tregs. TIM-3^+^ Tregs from human HNSCC also show an effector-like phenotype with highly robust expression of CTLA-4, PD-1, CD39, and IFN-γ receptors. Exogenous IFN-γ treatment can partially reverse the suppressive function of TIM-3^+^ TI-Tregs. Anti-PD-1 immunotherapy downregulates TIM-3 expression on Tregs isolated from HNSCC in rats and mice, which reverses the suppressive function of HNSCC TI-Tregs [191]. TIM-3 can be upregulated on CD8 T cells and Tregs in tumours treated with RT and PD-L1 blockades. Treatment with anti-TIM-3 administered with anti-PD-L1 and RT concurrently led to significant tumour growth delay, enhanced T-cell cytotoxicity, decreased Tregs levels, and the improved survival of orthotopic models of HNSCC. However, targeting Tregs depletion restored antitumour immunity in mice treated with radiotherapy (RT) and dual-ICB and resulted in tumour rejection and the induction of immunologic memory [192]. In a transgenic HNSCC mouse model, a blockade of TIM-3 by the anti-TIM-3 mAb induced a reduction in Tregs. Meanwhile, the population of TIM-3^+^ Tregs was also decreased. The increased IFN-γ^+^ CD8^+^ T cells in the anti-TIM-3-treated mice showed that the antitumour immune response is enhanced through the suppression of these negative immune factors [193]. The prognosis of follicular lymphoma (FL) patients is suspected to be influenced by TI-Tregs, which comprise activated ICOS^+^ Tregs that are able to inhibit not only conventional T cells but also FL B cells, which are able to express ICOSL and generate Tregs expressing ICOS. These Tregs were associated with ICOS/ICOSL engagement and were abrogated by antagonist anti-ICOS and anti-ICOSL antibodies [194, 195] (Table 1).

**Table 1:** The combination molecules on Tregs of targeted drugs in clinical trials

Targets	drugs	Cancer type	NCT
CD25	Basiliximab	T-Cell and NK-Cell Non-Hodgkin Lymphoma	NCT02342782
	Basiliximab	Recurrent Adult Hodgkin Lymphoma	NCT01476839
	Daclizumab	Hodgkin Lymphoma	NCT01468311
	Daclizumab	Melanoma	NCT00847106
CTLA-4	Tremelimumab	Cutaneous Melanoma	NCT04274816
	Tremelimumab	Malignant Mesothelioma	NCT01655888
	Tremelimumab	Colorectal Neoplasms Melanoma Prostatic Neoplasms Renal Cell Carcinoma Neoplasms Patients Who Have/Have Had Tumors	NCT00378482
	Tremelimumab	Malignant Mesothelioma	NCT01649024
	Ipilimumab	Melanoma	NCT02027935
	Ipilimumab	Prostate Cancer	NCT01804465
	Ipilimumab	HNSCC	NCT04080804
	Ipilimumab	Sarcoma Wilm’s Tumor Lymphoma Neuroblastoma	NCT01445379
	Ipilimumab	Melanoma	NCT00972933
	Ipilimumab	Pancreatic Cancer	NCT00112580
	Ipilimumab	Extensive Stage Small Cell Lung Cancer	NCT01331525
	CP-675,206	HCC	NCT01008358
	CP-675,206	Melanoma	NCT00431275
	BCD-145	Melanoma	NCT03472027
	ADU-1604	Metastatic Melanoma	NCT03674502
GITR	MK-4166	Glioblastoma	NCT03707457
	BMS-986156	MetastaticMalignant Solid Neoplasm	NCT04021043
	GWN323	Solid Tumors Lymphomas	NCT02740270
	TRX518	Unresectable Stage III or Stage IV Malignant Melanoma or Other Solid Tumor Malignancies	NCT01239134
LAG-3	Sym022	Metastatic Cancer Solid Tumor Lymphoma	NCT03489369
	anti-LAG-3	Multiple Myeloma Relapsed Refractory Multiple Myeloma	NCT04150965
	anti-LAG-3	Microsatellite Unstable Colorectal Cancer Microsatellite Stable Colorectal Cancer Mismatch Repair Proficient Colorectal Cancer Mismatch Repair Deficient Colorectal Cancer	NCT02060188
	BMS-986016	Glioblastoma Recurrent Brain Neoplasm	NCT02658981
	BMS-986016	Hematologic Neoplasms	NCT02061761
	Relatlimab	•Neoplasms by Site	NCT01968109
	Relatlimab	HNSCC	NCT04080804
	Relatlimab	Gastroesophageal Cancer	NCT03610711
	Relatlimab	Chordoma Locally Advanced Chordoma Metastatic Chordoma Unresectable Chordoma	NCT03623854
	Relatlimab	Various Advanced Cancer	NCT02488759
	Relatlimab	Advanced Cancer	NCT03459222
	Relatlimab	Melanoma	NCT03743766
	Relatlimab	Cancer	NCT02966548
	Relatlimab	Gastric Cancer Esophageal Cancer GastroEsophageal Cancer	NCT03044613
	Relatlimab	Microsatellite Stable (MSS) Colorectal Adenocarcinomas Colorectal Adenocarcinoma	NCT03642067
	REGN3767	Malignancies	NCT03005782
	Sym022	Metastatic Cancer Solid Tumor Lymphoma	NCT03311412
	TSR-033	Advanced or Metastatic Solid Tumors	NCT02817633
	TSR-033	Advanced Solid Tumors Antibodies Immunotherapy Colorectal Cancer	NCT03250832
	BMS-986213	Gastric Cancer Cancer of the Stomach Esophagogastric Junction	NCT03662659
TIGIT	BGB-A1217	Metastatic Solid Tumors	NCT04047862
	MTIG7192A	Non-small Cell Lung Cancer	NCT03563716
	Tiragolumab	Small Cell Lung Cancer	NCT04256421
	Tiragolumab	Non-Small Cell Lung Cancer	NCT04294810
OX40	anti-OX40	Head and Neck Cancer	NCT02274155
	anti-OX40	Advanced Cancer	NCT01644968
	anti-OX40	Metastatic Prostate Cancer Cancer of the Prostate Prostate Cancer	NCT01303705
	PF-04518600	Clear Cell Renal Cell Carcinoma Metastatic Renal Cell Cancer Recurrent Renal Cell Carcinoma Stage IV Renal Cell Cancer	NCT03092856
	PF-04518600	Stage III Breast Cancer Stage IIIA Breast Cancer Stage IIIB Breast Cancer Stage IIIC Breast Cancer Stage IV Breast Cancer Invasive Breast Carcinoma Recurrent Breast Carcinoma Triple-Negative Breast Carcinoma	NCT03971409
	PF-04518600	Advanced Malignant Solid Neoplasm Castration-Resistant Prostate Carcinoma Malignant Neoplasm Malignant Solid Neoplasm Metastatic Malignant Solid Neoplasm Metastatic Prostate Carcinoma Prostate Carcinoma Metastatic in the Bone Refractory Malignant Solid Neoplasm Stage IV Prostate Cancer AJCC v8 Stage IVA Prostate Cancer AJCC v8 Stage IVB Prostate Cancer AJCC v8	NCT03217747
	PF-04518600	Recurrent Acute Myeloid Leukemia Refractory Acute Myeloid Leukemia	NCT03390296
	MEDI6469	Colorectal Neoplasms	NCT02559024
	MEDI6469	Metastatic Breast Cancer Lung Metastases Liver Metastases	NCT01862900
	BMS 986178	B-Cell Non-Hodgkin Lymphoma Grade 1 Follicular Lymphoma Grade 2 Follicular Lymphoma Grade 3a Follicular Lymphoma Lymphoplasmacytic Lymphoma Mantle Cell Lymphoma Marginal Zone Lymphoma Small Lymphocytic Lymphoma	NCT03410901
	BMS 986178	Advanced Malignant Solid Neoplasm Extracranial Solid Neoplasm Metastatic Malignant Solid Neoplasm	NCT03831295
	MOXR0916	Neoplasms	NCT02410512
	BGB-A445	Advanced Solid Tumor	NCT04215978
ICOS	MEDI-570	Follicular T-Cell Lymphoma Grade 1 Follicular Lymphoma Grade 2 Follicular Lymphoma Grade 3a Follicular Lymphoma Mature T-Cell and NK-Cell Non-Hodgkin Lymphoma Recurrent Angioimmunoblastic T-Cell Lymphoma Recurrent Follicular Lymphoma Recurrent Mature T- Cell and NK-Cell Non-Hodgkin Lymphoma Recurrent Mycosis Fungoides Recurrent Primary Cutaneous T-Cell Non-Hodgkin Lymphoma	NCT02520791
	KY1044	Squamous Cell Carcinoma of Head and Neck Non-small Cell Lung Cancer Hepatocellular Carcinoma Esophageal Cancer Gastric Cancer Melanoma Renal Cell Carcinoma Pancreatic Cancer Cervical Cancer Triple Negative Breast Cancer Advanced Cancer	NCT03829501
CCR4	Mogamulizumab	Stage IB-IIB Cutaneous T-Cell Lymphoma	NCT04128072
	Mogamulizumab	Gastric Cancer Esophageal Cancer Lung Cancer Renal Cancer Oral Cancer	NCT02946671
	KW-0761	Cutaneous T-Cell Lymphoma	NCT01728805
	KW-0761	Adult T-cell Leukemia-Lymphoma	NCT01626664
	KW-0761	Peripheral T-cell Lymphoma Cutaneous T-cell Lymphoma	NCT01226472
	KW-0761	Peripheral T-Cell Lymphoma	NCT01611142
	KW-0761	Peripheral T-Cell Lymphoma	NCT00888927

### Skewing Tregs towards anti-tumour immunity phenotype

Despite the opposite roles of T-bet and Foxp3 in the immune system and tumour biology, recent studies have demonstrated the presence of CD4^+^ T cells expressing both T-bet and Foxp3. T-bet^+^Foxp3^+^CD4^+^ T cells mediated by the immunosuppressive cytokine TGF-β accumulate in the lungs of tumour-bearing mice and are characterized as Th1-like Tregs. The conversion of IFN-γ-producing antitumoural T-bet^+^Th1 CD4^+^ T cells into immunosuppressive T-bet and Foxp3-PD-1 coexpressing Tregs may represent an additional and important mechanism of the TGF-β-mediated blockade of antitumour immunity [196]. The main factors driving the differentiation of Tregs towards a pro-inflammatory phenotype include IL-12 for Th1-like Tregs and IL-6 for Th17-type Tregs. Importantly, the blockade of IFN-γ partially restores the suppressive function of Tregs [197]. The signalling events driving the generation of human Th1-Tregs depend on the PI3K/AKT/Foxo1/3 signalling cascade, which is the major pathway involved in IFN-γ secretion by human Tregs [198]. Moreover, Foxo1 can be recruited to a regulatory element upstream of the transcriptional start site of the IFNG gene. Treg-specific deletion of Foxo1 leads to upregulation of IFNG gene expression and increased IFN-γ^+^ Tregs [199, 200]. The E3 ubiquitin ligase VHL can regulate HIF-1α to maintain the stability and suppressive capacity of Tregs. VHL-deficient Tregs failed to prevent colitis induction but were converted into Th1-like effector T cells with excessive IFN-γ production. VHL intrinsically orchestrated this conversion under both steady and inflammatory conditions followed by Foxp3 downregulation, which was reversed by IFN-γ deficiency. Augmented HIF-1α-induced glycolytic reprogramming was required for IFN-γ production. Furthermore, HIF-1α bound directly to the *IFNG* promoter. Knockdown or knockout of HIF-1α reversed the increase in IFN-γ by VHL-deficient Tregs and restored their suppressive capacities in vivo [201].

### Targeting other molecules on Tregs to suppress Tregs functions

NRP1 plays an important role in the stability and function of intratumoural Tregs. The NRP1 antagonist Fc (AAG)-TPP11, generated by fusion of the NRP1-specific binding peptide TPP11 with the C-terminus of an effector function-deficient immunoglobulin Fc (AAG) variant, inhibits intratumoural NRP1^+^ Tregs function and stability, which triggers the internalization of NRP1, reduces its surface expression, and thereby inhibits the suppressive function of Tregs [202]. Treg-restricted NRP1 deletion results in profound tumour resistance due to Tregs functional fragility. A high percentage of intratumoural NRP1^+^ Tregs correlated with poor prognosis for melanoma and HNSCC patients. However, a high proportion of intratumoural Nrp1^−/−^ Tregs produced IFN-γ, driving the fragility of surrounding wild-type Tregs, boosting antitumour immunity, and facilitating tumour clearance, which is required for the response to anti-PD-1 [71]. The specific deletion of the deubiquitinase POH1 gene in T cells compromised the development of mature T cells, especially CD4^+^Foxp3^+^ Tregs. Furthermore, POH1 deficiency significantly attenuated the transition of CD25^+^ Tregs precursors into Foxp3^+^ Tregs and was accompanied by downregulation of IL-2-STAT5 signalling [203]. Idelalisib is a highly selective PI3K (PI3Kδ) isoform-specific inhibitor effective in relapsed/refractory CLL and follicular lymphoma. Compared with CD4^+^ and CD8^+^ effector T cells, human Tregs are highly susceptible to PI3Kδ inactivation using idelalisib, as evident from its effects on anti-CD3/CD28/CD2-induced proliferation and the level of AKT and NF-kB phosphorylation. Additionally, Tregs treated with idelalisib can show significantly altered phenotypes and downregulation of their suppressive function [204]. Inactivation of PI(3) K p110δ disrupts Treg-mediated immune tolerance to cancer. In mice, p110δ inactivation protected against a broad range of cancers, including non-haematological solid tumours. p110δ inactivation in Tregs unleashes CD8^+^ cytotoxic T cells and induces tumour regression. Thus, p110δ inhibitors can attenuate tumour-induced immune tolerance and should be considered for wider use in oncology [205].

## Conclusions and future perspectives

The immunosuppressive activity of Tregs in tumours is a major obstacle to effective antitumour immunity. Combining Treg-targeting therapies with other approaches, such as IC blockades, immune-agonists, tumour vaccines, radiotherapy, and chemotherapy, provides a synergistic antitumour effect. However, due to the production of cytokines, chemokines, and foreign body-mediated reprogramming in the tumour microenvironment, the functional evaluation of Tregs in tumour tissues is complex and difficult to determine. Early tumour immunotherapy targeting Tregs mainly focused on clearance, but the effect was not ideal. There may be several reasons for this outcome. On the one hand, when removing Tregs, normal effector T cells are also removed, which leads to a significant decline in the body’s antitumour immunity. On the other hand, Tregs clearance is only temporary, and the Tregs levels in the body will soon return to the level before clearance. Therefore, the idea of immunotherapy targeting Tregs should be changed from clearing to controlling the number and inducing the functional differentiation of Tregs towards Th1-like Tregs. To design better therapeutic options targeted to Tregs in cancer, it is necessary to explore the signalling pathways that govern the acquisition of specific effector characteristics by Tregs. In the future, a better option will be the induction of redifferentiation or reprogramming of Tregs in tumours towards Th1-like Tregs, which produce IFN-γ and IL-12 to kill tumour cells. In addition, targeting Tregs in combination with other cancer therapies will be another good option. However, the pattern of these combinations during treatment requires a deeper study into the dynamics of the tumour microenvironment to determine how to obtain optimal combinations. Therefore, the roles and functions of Tregs need to be further studied to reach it’s the potential of Tregs as immunotherapeutic targets and provide a new strategies for tumour immunotherapy.

## Supplementary information

**Additional file 1.**

## Data Availability

Not applicable.

## Abbreviations

Tregs: Regulatory T cells
Foxp3: Forkhead box protein p3
TME: Tumour microenvironment
TGF-β: Transforming growth factor-beta
CTLs: Cytotoxic T lymphocytes
Blimp1: B lymphocyte induced maturation protein 1
DCs: Dendritic cells
CTLA-4: Cytotoxic T lymphocyte-associated antigen-4
nTregs: Naturally occurring Tregs
iTregs: Inducible Tregs
BTLA: B and T lymphocyte attenuator
BM: Bone marrow
scRNA-seq: Single-cell RNA-sequencing
Teff: Effector T cells
GZM: Granzyme
SREBP1: Sterol regulatory element-binding protein 1
TAMs: Tumour-associated macrophages
FABPs: Fatty acid binding proteins
VAT: Visceral adipose tissue
TET: Ten-eleven translocation
mTORC: Mammalian target of rapamycin complex
PPARβ: Peroxisome proliferator-activated receptor-beta
LKB1: Liver Kinase B1
TSDR: TREG-specific demethylated region
CNS: Conserved non-coding sequence
HIC1: Hypermethylated in cancer 1
EZH2: Enhancer of zeste homolog 2
SENP3: SUMO-specific protease 3
PRMT: Protein arginine methytransferase
CRC: Colorectal cancer
LTα1β2: Lymphotoxin alpha beta 2
LECs: Lymphatic endothelial cells
GARP: Glycoprotein-A repetitions predominant
TDLN: Tumor-draining lymph nodes
S1P1: Sphingosine-1-phosphate receptor 1
ETBF: Enterotoxigenic *Bacteroides fragilis*
HDC: Histamine dihydrochloride
LHME: Leukemic hematopoietic microenvironment
ADCC: Antibody-dependent cell mediated cytotoxicity
ADCP: ADCC and cellular phagocytosis
CLL: Chronic lymphocytic leukemia
RT: Radiotherapy

## References

[1] van der Veeken J, Gonzalez AJ, Cho H, Arvey A, Hemmers S, Leslie CS, Rudensky AY (2016). Memory of inflammation in regulatory T cells. Cell.

[2] Newton R, Priyadharshini B, Turka LA (2016). Immunometabolism of regulatory T cells. Nat Immunol.

[3] Li MO, Rudensky AY (2016). T cell receptor signalling in the control of regulatory T cell differentiation and function. Nat Rev Immunol.

[4] Sakaguchi S, Mikami N, Wing JB, Tanaka A, Ichiyama K, Ohkura N. Regulatory T cells and human disease. Annu Rev Immunol. 2020;38:541–66. 10.1146/annurev-immunol-042718-041717 32017635

[5] Toker A, Ohashi PS (2019). Expression of costimulatory and inhibitory receptors in FoxP3(+) regulatory T cells within the tumor microenvironment: implications for combination immunotherapy approaches. Adv Cancer Res.

[6] Campbell C, Rudensky A (2020). Roles of regulatory T cells in tissue pathophysiology and metabolism. Cell Metab.

[7] Cuadrado E, van den Biggelaar M, de Kivit S, Chen YY, Slot M, Doubal I, Meijer A, van Lier RAW, Borst J, Amsen D (2018). Proteomic analyses of human regulatory T cells reveal adaptations in signaling pathways that protect cellular identity. Immunity.

[8] Gershon RK, Kondo K (1970). Cell interactions in the induction of tolerance: the role of thymic lymphocytes. Immunology.

[9] Gershon RK, Kondo K (1971). Infectious immunological tolerance. Immunology.

[10] Gershon RK, Cohen P, Hencin R, Liebhaber SA (1972). Suppressor T cells. J Immunol.

[11] Moller G (1988). Do suppressor T cells exist?. Scand J Immunol.

[12] Groux H, O'Garra A, Bigler M, Rouleau M, Antonenko S, de Vries JE, Roncarolo MG (1997). A CD4+ T-cell subset inhibits antigen-specific T-cell responses and prevents colitis. Nature.

[13] Sakaguchi S, Wing K, Miyara M (2007). Regulatory T cells - a brief history and perspective. Eur J Immunol.

[14] Dominguez-Villar M, Hafler DA. Regulatory T cells in autoimmune disease. Nat Immunol. 2018;19(7):665–73. 10.1038/s41590-018-0120-4 PMC7882196 29925983

[15] Josefowicz SZ, Lu LF, Rudensky AY (2012). Regulatory T cells: mechanisms of differentiation and function. Annu Rev Immunol.

[16] Sakaguchi S, Miyara M, Costantino CM, Hafler DA (2010). FOXP3+ regulatory T cells in the human immune system. Nat Rev Immunol.

[17] Sakaguchi S, Yamaguchi T, Nomura T, Ono M (2008). Regulatory T cells and immune tolerance. Cell.

[18] Samstein RM, Arvey A, Josefowicz SZ, Peng X, Reynolds A, Sandstrom R, Neph S, Sabo P, Kim JM, Liao W (2012). Foxp3 exploits a pre-existent enhancer landscape for regulatory T cell lineage specification. Cell.

[19] Lu L, Barbi J, Pan F (2017). The regulation of immune tolerance by FOXP3. Nat Rev Immunol.

[20] Tanaka A, Sakaguchi S (2017). Regulatory T cells in cancer immunotherapy. Cell Res.

[21] Hori S, Nomura T, Sakaguchi S (2003). Control of regulatory T cell development by the transcription factor Foxp3. Science.

[22] Fontenot JD, Gavin MA, Rudensky AY (2003). Foxp3 programs the development and function of CD4+CD25+ regulatory T cells. Nat Immunol.

[23] Fontenot JD, Rasmussen JP, Williams LM, Dooley JL, Farr AG, Rudensky AY (2005). Regulatory T cell lineage specification by the forkhead transcription factor foxp3. Immunity.

[24] Schubert LA, Jeffery E, Zhang Y, Ramsdell F, Ziegler SF (2001). Scurfin (FOXP3) acts as a repressor of transcription and regulates T cell activation. J Biol Chem.

[25] Pillai V, Ortega SB, Wang CK, Karandikar NJ (2007). Transient regulatory T-cells: a state attained by all activated human T-cells. Clin Immunol.

[26] Spolski R, Li P, Leonard WJ. Biology and regulation of IL-2: from molecular mechanisms to human therapy. Nat Rev Immunol. 2018;18(10):648–59. 10.1038/s41577-018-0046-y 30089912

[27] Nishikawa H, Sakaguchi S (2014). Regulatory T cells in cancer immunotherapy. Curr Opin Immunol.

[28] Colombo MP, Piconese S (2007). Regulatory-T-cell inhibition versus depletion: the right choice in cancer immunotherapy. Nat Rev Cancer.

[29] Sullivan JA, Tomita Y, Jankowska-Gan E, Lema DA, Arvedson MP, Nair A, Bracamonte-Baran W, Zhou Y, Meyer KK, Zhong W (2020). Treg-cell-derived IL-35-coated extracellular vesicles promote infectious tolerance. Cell Rep.

[30] Sawant DV, Yano H, Chikina M, Zhang Q, Liao M, Liu C, Callahan DJ, Sun Z, Sun T, Tabib T (2019). Adaptive plasticity of IL-10(+) and IL-35(+) Treg cells cooperatively promotes tumor T cell exhaustion. Nat Immunol.

[31] Wing JB, Tanaka A, Sakaguchi S (2019). Human FOXP3(+) regulatory T cell heterogeneity and function in autoimmunity and Cancer. Immunity.

[32] Kanamori M, Nakatsukasa H, Okada M, Lu Q, Yoshimura A (2016). Induced regulatory T cells: their development, stability, and applications. Trends Immunol.

[33] Leonard JD, Gilmore DC, Dileepan T, Nawrocka WI, Chao JL, Schoenbach MH, Jenkins MK, Adams EJ, Savage PA (2017). Identification of natural regulatory T cell epitopes reveals convergence on a dominant autoantigen. Immunity.

[34] Vasanthakumar A, Liao Y, Teh P, Pascutti MF, Oja AE, Garnham AL, Gloury R, Tempany JC, Sidwell T, Cuadrado E (2017). The TNF receptor superfamily-NF-kappaB Axis is critical to maintain effector regulatory T cells in lymphoid and non-lymphoid tissues. Cell Rep.

[35] Oh H, Grinberg-Bleyer Y, Liao W, Maloney D, Wang P, Wu Z, Wang J, Bhatt DM, Heise N, Schmid RM (2017). An NF-kappaB transcription-factor-dependent lineage-specific transcriptional program promotes regulatory T cell identity and function. Immunity.

[36] Grinberg-Bleyer Y, Oh H, Desrichard A, Bhatt DM, Caron R, Chan TA, Schmid RM, Klein U, Hayden MS, Ghosh S (2017). NF-kappaB c-Rel is crucial for the regulatory T cell immune checkpoint in Cancer. Cell.

[37] Owen DL, Mahmud SA, Sjaastad LE, Williams JB, Spanier JA, Simeonov DR, Ruscher R, Huang W, Proekt I, Miller CN (2019). Thymic regulatory T cells arise via two distinct developmental programs. Nat Immunol.

[38] Wang L, Simons DL, Lu X, Tu TY, Solomon S, Wang R, Rosario A, Avalos C, Schmolze D, Yim J (2019). Connecting blood and intratumoral Treg cell activity in predicting future relapse in breast cancer. Nat Immunol.

[39] Ahmadzadeh M, Pasetto A, Jia L, Deniger DC, Stevanovic S, Robbins PF, Rosenberg SA. Tumor-infiltrating human CD4(+) regulatory T cells display a distinct TCR repertoire and exhibit tumor and neoantigen reactivity. Sci Immunol. 2019;4(31):eaao4310. 10.1126/sciimmunol.aao4310 PMC6685542 30635355

[40] Downs-Canner S, Berkey S, Delgoffe GM, Edwards RP, Curiel T, Odunsi K, Bartlett DL, Obermajer N (2017). Suppressive IL-17A(+)Foxp3(+) and ex-Th17 IL-17A(neg)Foxp3(+) Treg cells are a source of tumour-associated Treg cells. Nat Commun.

[41] Delacher M, Imbusch CD, Hotz-Wagenblatt A, Mallm JP, Bauer K, Simon M, Riegel D, Rendeiro AF, Bittner S, Sanderink L (2020). Precursors for nonlymphoid-tissue Treg cells reside in secondary lymphoid organs and are programmed by the transcription factor BATF. Immunity.

[42] Jang SW, Hwang SS, Kim HS, Kim MK, Lee WH, Hwang SU, Gwak J, Yew SK, Flavell RA, Lee GR (2019). Homeobox protein Hhex negatively regulates Treg cells by inhibiting Foxp3 expression and function. Proc Natl Acad Sci U S A.

[43] Toomer KH, Malek TR. Cytokine signaling in the development and homeostasis of regulatory T cells. Cold Spring Harb Perspect Biol. 2018;10(3):a028597. 10.1101/cshperspect.a028597 PMC5830895 28620098

[44] Maj T, Wang W, Crespo J, Zhang H, Wang W, Wei S, Zhao L, Vatan L, Shao I, Szeliga W (2017). Oxidative stress controls regulatory T cell apoptosis and suppressor activity and PD-L1-blockade resistance in tumor. Nat Immunol.

[45] Wei X, Zhang J, Gu Q, Huang M, Zhang W, Guo J, Zhou X (2017). Reciprocal expression of IL-35 and IL-10 defines two distinct effector Treg subsets that are required for maintenance of immune tolerance. Cell Rep.

[46] Budhu S, Schaer DA, Li Y, Toledo-Crow R, Panageas K, Yang X, Zhong H, Houghton AN, Silverstein SC, Merghoub T, Wolchok JD. Blockade of surface-bound TGF-beta on regulatory T cells abrogates suppression of effector T cell function in the tumor microenvironment. Sci Signal. 2017;10(494):eaak9702. 10.1126/scisignal.aak9702 PMC5851440 28851824

[47] Kalia V, Penny LA, Yuzefpolskiy Y, Baumann FM, Sarkar S (2015). Quiescence of memory CD8(+) T cells is mediated by regulatory T cells through inhibitory receptor CTLA-4. Immunity.

[48] Sarhan D, Hippen KL, Lemire A, Hying S, Luo X, Lenvik T, Curtsinger J, Davis Z, Zhang B, Cooley S (2018). Adaptive NK cells resist regulatory T-cell suppression driven by IL37. Cancer Immunol Res.

[49] Liu C, Chikina M, Deshpande R, Menk AV, Wang T, Tabib T, Brunazzi EA, Vignali KM, Sun M, Stolz DB (2019). Treg cells promote the SREBP1-dependent metabolic fitness of tumor-promoting macrophages via repression of CD8(+) T cell-derived interferon-gamma. Immunity.

[50] Carmenate T, Ortiz Y, Enamorado M, Garcia-Martinez K, Avellanet J, Moreno E, Graca L, Leon K (2018). Blocking IL-2 signal in vivo with an IL-2 antagonist reduces tumor growth through the control of regulatory T cells. J Immunol.

[51] Ohta A, Kini R, Ohta A, Subramanian M, Madasu M, Sitkovsky M (2012). The development and immunosuppressive functions of CD4(+) CD25(+) FoxP3(+) regulatory T cells are under influence of the adenosine-A2A adenosine receptor pathway. Front Immunol.

[52] Park YJ, Ryu H, Choi G, Kim BS, Hwang ES, Kim HS, Chung Y (2019). IL-27 confers a protumorigenic activity of regulatory T cells via CD39. Proc Natl Acad Sci U S A.

[53] Belle L, Agle K, Zhou V, Yin-Yuan C, Komorowski R, Eastwood D, Logan B, Sun J, Ghilardi N, Cua D (2016). Blockade of interleukin-27 signaling reduces GVHD in mice by augmenting Treg reconstitution and stabilizing Foxp3 expression. Blood.

[54] Ihara F, Sakurai D, Takami M, Kamata T, Kunii N, Yamasaki K, Iinuma T, Nakayama T, Motohashi S, Okamoto Y (2019). Regulatory T cells induce CD4(−) NKT cell anergy and suppress NKT cell cytotoxic function. Cancer Immunol Immunother.

[55] Liu J, Zhang H, Jia L, Sun H (2015). Effects of Treg cells and IDO on human epithelial ovarian cancer cells under hypoxic conditions. Mol Med Rep.

[56] Ghiringhelli F, Puig PE, Roux S, Parcellier A, Schmitt E, Solary E, Kroemer G, Martin F, Chauffert B, Zitvogel L (2005). Tumor cells convert immature myeloid dendritic cells into TGF-beta-secreting cells inducing CD4+CD25+ regulatory T cell proliferation. J Exp Med.

[57] Morello S, Pinto A, Blandizzi C, Antonioli L (2016). Myeloid cells in the tumor microenvironment: role of adenosine. Oncoimmunology.

[58] Huang B, Pan PY, Li Q, Sato AI, Levy DE, Bromberg J, Divino CM, Chen SH (2006). Gr-1+CD115+ immature myeloid suppressor cells mediate the development of tumor-induced T regulatory cells and T-cell anergy in tumor-bearing host. Cancer Res.

[59] Hoechst B, Ormandy LA, Ballmaier M, Lehner F, Kruger C, Manns MP, Greten TF, Korangy F (2008). A new population of myeloid-derived suppressor cells in hepatocellular carcinoma patients induces CD4(+)CD25(+)Foxp3(+) T cells. Gastroenterology.

[60] Platten M, Nollen EAA, Rohrig UF, Fallarino F, Opitz CA (2019). Tryptophan metabolism as a common therapeutic target in cancer, neurodegeneration and beyond. Nat Rev Drug Discov.

[61] Moon YW, Hajjar J, Hwu P, Naing A (2015). Targeting the indoleamine 2,3-dioxygenase pathway in cancer. J Immunother Cancer.

[62] Jitschin R, Braun M, Buttner M, Dettmer-Wilde K, Bricks J, Berger J, Eckart MJ, Krause SW, Oefner PJ, Le Blanc K (2014). CLL-cells induce IDOhi CD14+HLA-DRlo myeloid-derived suppressor cells that inhibit T-cell responses and promote TRegs. Blood.

[63] Pan PY, Ma G, Weber KJ, Ozao-Choy J, Wang G, Yin B, Divino CM, Chen SH (2010). Immune stimulatory receptor CD40 is required for T-cell suppression and T regulatory cell activation mediated by myeloid-derived suppressor cells in cancer. Cancer Res.

[64] Byrne KT, Vonderheide RH (2016). CD40 stimulation obviates innate sensors and drives T cell immunity in Cancer. Cell Rep.

[65] Yang R, Cai Z, Zhang Y, Yutzy WH, Roby KF, Roden RB (2006). CD80 in immune suppression by mouse ovarian carcinoma-associated gr-1+CD11b+ myeloid cells. Cancer Res.

[66] Lee CR, Kwak Y, Yang T, Han JH, Park SH, Ye MB, Lee W, Sim KY, Kang JA, Kim YC (2016). Myeloid-derived suppressor cells are controlled by regulatory T cells via TGF-beta during murine colitis. Cell Rep.

[67] Kochetkova I, Golden S, Holderness K, Callis G, Pascual DW (2010). IL-35 stimulation of CD39+ regulatory T cells confers protection against collagen II-induced arthritis via the production of IL-10. J Immunol.

[68] Seyerl M, Kirchberger S, Majdic O, Seipelt J, Jindra C, Schrauf C, Stockl J (2010). Human rhinoviruses induce IL-35-producing Treg via induction of B7-H1 (CD274) and sialoadhesin (CD169) on DC. Eur J Immunol.

[69] Fujimura T, Kambayashi Y, Aiba S (2012). Crosstalk between regulatory T cells (Tregs) and myeloid derived suppressor cells (MDSCs) during melanoma growth. Oncoimmunology.

[70] Di Pilato M, Kim EY, Cadilha BL, Prussmann JN, Nasrallah MN, Seruggia D, Usmani SM, Misale S, Zappulli V, Carrizosa E (2019). Targeting the CBM complex causes Treg cells to prime tumours for immune checkpoint therapy. Nature.

[71] Overacre-Delgoffe AE, Chikina M, Dadey RE, Yano H, Brunazzi EA, Shayan G, Horne W, Moskovitz JM, Kolls JK, Sander C (2017). Interferon-gamma drives Treg fragility to promote anti-tumor immunity. Cell.

[72] Noval Rivas M, Burton OT, Wise P, Charbonnier LM, Georgiev P, Oettgen HC, Rachid R, Chatila TA (2015). Regulatory T cell reprogramming toward a Th2-cell-like lineage impairs oral tolerance and promotes food allergy. Immunity.

[73] Krishnamoorthy N, Khare A, Oriss TB, Raundhal M, Morse C, Yarlagadda M, Wenzel SE, Moore ML, Peebles RS, Ray A, Ray P (2012). Early infection with respiratory syncytial virus impairs regulatory T cell function and increases susceptibility to allergic asthma. Nat Med.

[74] Halim L, Romano M, McGregor R, Correa I, Pavlidis P, Grageda N, Hoong SJ, Yuksel M, Jassem W, Hannen RF (2017). An atlas of human regulatory T helper-like cells reveals features of Th2-like Tregs that support a tumorigenic environment. Cell Rep.

[75] Ayyoub M, Deknuydt F, Raimbaud I, Dousset C, Leveque L, Bioley G, Valmori D (2009). Human memory FOXP3+ Tregs secrete IL-17 ex vivo and constitutively express the T(H)17 lineage-specific transcription factor RORgamma t. Proc Natl Acad Sci U S A.

[76] Voo KS, Wang YH, Santori FR, Boggiano C, Wang YH, Arima K, Bover L, Hanabuchi S, Khalili J, Marinova E (2009). Identification of IL-17-producing FOXP3+ regulatory T cells in humans. Proc Natl Acad Sci U S A.

[77] Beriou G, Costantino CM, Ashley CW, Yang L, Kuchroo VK, Baecher-Allan C, Hafler DA (2009). IL-17-producing human peripheral regulatory T cells retain suppressive function. Blood.

[78] Field CS, Baixauli F, Kyle RL, Puleston DJ, Cameron AM, Sanin DE, Hippen KL, Loschi M, Thangavelu G, Corrado M (2020). Mitochondrial integrity regulated by lipid metabolism is a cell-intrinsic checkpoint for Treg suppressive function. Cell Metab.

[79] Hang S, Paik D, Yao L, Kim E, Trinath J, Lu J, Ha S, Nelson BN, Kelly SP, Wu L (2019). Bile acid metabolites control TH17 and Treg cell differentiation. Nature.

[80] Song X, Sun X, Oh SF, Wu M, Zhang Y, Zheng W, Geva-Zatorsky N, Jupp R, Mathis D, Benoist C, Kasper DL (2020). Microbial bile acid metabolites modulate gut RORgamma(+) regulatory T cell homeostasis. Nature.

[81] Britton GJ, Contijoch EJ, Mogno I, Vennaro OH, Llewellyn SR, Ng R, Li Z, Mortha A, Merad M, Das A (2019). Microbiotas from humans with inflammatory bowel disease Alter the balance of gut Th17 and RORgammat(+) regulatory T cells and exacerbate colitis in mice. Immunity.

[82] Pacella I, Procaccini C, Focaccetti C, Miacci S, Timperi E, Faicchia D, Severa M, Rizzo F, Coccia EM, Bonacina F (2018). Fatty acid metabolism complements glycolysis in the selective regulatory T cell expansion during tumor growth. Proc Natl Acad Sci U S A.

[83] Sundstrom P, Stenstad H, Langenes V, Ahlmanner F, Theander L, Ndah TG, Fredin K, Borjesson L, Gustavsson B, Bastid J, Quiding-Jarbrink M (2016). Regulatory T cells from Colon Cancer patients inhibit effector T-cell migration through an adenosine-dependent mechanism. Cancer Immunol Res.

[84] Vasanthakumar A, Chisanga D, Blume J, Gloury R, Britt K, Henstridge DC, Zhan Y, Torres SV, Liene S, Collins N, et al. Sex-specific adipose tissue imprinting of regulatory T cells. Nature. 2020;579(7800):581–85. 10.1038/s41586-020-2040-3 PMC7241647 32103173

[85] Fu Z, Ye J, Dean JW, Bostick JW, Weinberg SE, Xiong L, Oliff KN, Chen ZE, Avram D, Chandel NS, Zhou L (2019). Requirement of mitochondrial transcription factor a in tissue-resident regulatory T cell maintenance and function. Cell Rep.

[86] Weinberg SE, Singer BD, Steinert EM, Martinez CA, Mehta MM, Martinez-Reyes I, Gao P, Helmin KA, Abdala-Valencia H, Sena LA (2019). Mitochondrial complex III is essential for suppressive function of regulatory T cells. Nature.

[87] Liu X, Mo W, Ye J, Li L, Zhang Y, Hsueh EC, Hoft DF, Peng G (2018). Regulatory T cells trigger effector T cell DNA damage and senescence caused by metabolic competition. Nat Commun.

[88] Charbonnier LM, Cui Y, Stephen-Victor E, Harb H, Lopez D, Bleesing JJ, Garcia-Lloret MI, Chen K, Ozen A, Carmeliet P (2019). Functional reprogramming of regulatory T cells in the absence of Foxp3. Nat Immunol.

[89] Wang H, Franco F, Tsui YC, Xie X, Trefny MP, Zappasodi R, Mohmood SR, Fernandez-Garcia J, Tsai CH, Schulze I (2020). CD36-mediated metabolic adaptation supports regulatory T cell survival and function in tumors. Nat Immunol.

[90] Pelgrom LR, Patente TA, Sergushichev A, Esaulova E, Otto F, Ozir-Fazalalikhan A, van der Zande HJP, van der Ham AJ, van der Stel S, Artyomov MN, Everts B (2019). LKB1 expressed in dendritic cells governs the development and expansion of thymus-derived regulatory T cells. Cell Res.

[91] Chen S, Fang L, Guo W, Zhou Y, Yu G, Li W, Dong K, Liu J, Luo Y, Wang B (2018). Control of Treg cell homeostasis and immune equilibrium by Lkb1 in dendritic cells. Nat Commun.

[92] Yang K, Blanco DB, Neale G, Vogel P, Avila J, Clish CB, Wu C, Shrestha S, Rankin S, Long L (2017). Homeostatic control of metabolic and functional fitness of Treg cells by LKB1 signalling. Nature.

[93] Tanaka S, Pfleger C, Lai JF, Roan F, Sun SC, Ziegler SF (2018). KAP1 regulates regulatory T cell function and proliferation in both Foxp3-dependent and -independent manners. Cell Rep.

[94] Chapman NM, Zeng H, Nguyen TM, Wang Y, Vogel P, Dhungana Y, Liu X, Neale G, Locasale JW, Chi H (2018). mTOR coordinates transcriptional programs and mitochondrial metabolism of activated Treg subsets to protect tissue homeostasis. Nat Commun.

[95] Yu X, Teng XL, Wang F, Zheng Y, Qu G, Zhou Y, Hu Z, Wu Z, Chang Y, Chen L (2018). Metabolic control of regulatory T cell stability and function by TRAF3IP3 at the lysosome. J Exp Med.

[96] Apostolidis SA, Rodriguez-Rodriguez N, Suarez-Fueyo A, Dioufa N, Ozcan E, Crispin JC, Tsokos MG, Tsokos GC (2016). Phosphatase PP2A is requisite for the function of regulatory T cells. Nat Immunol.

[97] Sun IH, Oh MH, Zhao L, Patel CH, Arwood ML, Xu W, Tam AJ, Blosser RL, Wen J, Powell JD (2018). mTOR complex 1 signaling regulates the generation and function of central and effector Foxp3(+) regulatory T cells. J Immunol.

[98] Gerriets VA, Kishton RJ, Johnson MO, Cohen S, Siska PJ, Nichols AG, Warmoes MO, de Cubas AA, MacIver NJ, Locasale JW (2016). Foxp3 and toll-like receptor signaling balance Treg cell anabolic metabolism for suppression. Nat Immunol.

[99] Li L, Liu X, Sanders KL, Edwards JL, Ye J, Si F, Gao A, Huang L, Hsueh EC, Ford DA (2019). TLR8-mediated metabolic control of human Treg function: a mechanistic target for Cancer immunotherapy. Cell Metab.

[100] Shi H, Chapman NM, Wen J, Guy C, Long L, Dhungana Y, Rankin S, Pelletier S, Vogel P, Wang H (2019). Amino acids license kinase mTORC1 activity and Treg cell function via small G proteins rag and Rheb. Immunity.

[101] Do MH, Wang X, Zhang X, Chou C, Nixon BG, Capistrano KJ, Peng M, Efeyan A, Sabatini DM, Li MO. Nutrient mTORC1 signaling underpins regulatory T cell control of immune tolerance. J Exp Med. 2020;217(1):e20190848. 10.1084/jem.20190848 PMC7037250 31649036

[102] Ng MSF, Roth TL, Mendoza VF, Marson A, Burt TD. Helios enhances the preferential differentiation of human fetal CD4(+) naive T cells into regulatory T cells. Sci Immunol. 2019;4(41):eaav5947. 10.1126/sciimmunol.aav5947 PMC7340007 31757834

[103] Kim HJ, Barnitz RA, Kreslavsky T, Brown FD, Moffett H, Lemieux ME, Kaygusuz Y, Meissner T, Holderried TA, Chan S (2015). Stable inhibitory activity of regulatory T cells requires the transcription factor Helios. Science.

[104] Yates K, Bi K, Haining WN, Cantor H, Kim HJ (2018). Comparative transcriptome analysis reveals distinct genetic modules associated with Helios expression in intratumoral regulatory T cells. Proc Natl Acad Sci U S A.

[105] Konopacki C, Pritykin Y, Rubtsov Y, Leslie CS, Rudensky AY (2019). Transcription factor Foxp1 regulates Foxp3 chromatin binding and coordinates regulatory T cell function. Nat Immunol.

[106] Shimazu Y, Shimazu Y, Hishizawa M, Hamaguchi M, Nagai Y, Sugino N, Fujii S, Kawahara M, Kadowaki N, Nishikawa H (2016). Hypomethylation of the Treg-specific Demethylated region in FOXP3 is a Hallmark of the regulatory T-cell subtype in adult T-cell leukemia. Cancer Immunol Res.

[107] Feng Y, Arvey A, Chinen T, van der Veeken J, Gasteiger G, Rudensky AY (2014). Control of the inheritance of regulatory T cell identity by a cis element in the Foxp3 locus. Cell.

[108] Garg G, Muschaweckh A, Moreno H, Vasanthakumar A, Floess S, Lepennetier G, Oellinger R, Zhan Y, Regen T, Hiltensperger M (2019). Blimp1 prevents methylation of Foxp3 and loss of regulatory T cell identity at sites of inflammation. Cell Rep.

[109] Feng Y, van der Veeken J, Shugay M, Putintseva EV, Osmanbeyoglu HU, Dikiy S, Hoyos BE, Moltedo B, Hemmers S, Treuting P (2015). A mechanism for expansion of regulatory T-cell repertoire and its role in self-tolerance. Nature.

[110] Ni X, Tao J, Barbi J, Chen Q, Park BV, Li Z, Zhang N, Lebid A, Ramaswamy A, Wei P (2018). YAP is essential for Treg-mediated suppression of antitumor immunity. Cancer Discov.

[111] Geng J, Yu S, Zhao H, Sun X, Li X, Wang P, Xiong X, Hong L, Xie C, Gao J (2017). The transcriptional coactivator TAZ regulates reciprocal differentiation of TH17 cells and Treg cells. Nat Immunol.

[112] Hibino S, Chikuma S, Kondo T, Ito M, Nakatsukasa H, Omata-Mise S, Yoshimura A (2018). Inhibition of Nr4a receptors enhances antitumor immunity by breaking Treg-mediated immune tolerance. Cancer Res.

[113] Castillo J, Wu E, Lowe C, Srinivasan S, McCord R, Wagle MC, Jayakar S, Edick MG, Eastham-Anderson J, Liu B (2019). CBP/p300 drives the differentiation of regulatory T cells through transcriptional and non-transcriptional mechanisms. Cancer Res.

[114] Becher J, Simula L, Volpe E, Procaccini C, La Rocca C, D'Acunzo P, Cianfanelli V, Strappazzon F, Caruana I, Nazio F (2018). AMBRA1 controls regulatory T-cell differentiation and homeostasis upstream of the FOXO3-FOXP3 Axis. Dev Cell.

[115] Wang D, Quiros J, Mahuron K, Pai CC, Ranzani V, Young A, Silveria S, Harwin T, Abnousian A, Pagani M (2018). Targeting EZH2 reprograms Intratumoral regulatory T cells to enhance Cancer immunity. Cell Rep.

[116] Yu X, Lao Y, Teng XL, Li S, Zhou Y, Wang F, Guo X, Deng S, Chang Y, Wu X (2018). SENP3 maintains the stability and function of regulatory T cells via BACH2 deSUMOylation. Nat Commun.

[117] Chinen T, Kannan AK, Levine AG, Fan X, Klein U, Zheng Y, Gasteiger G, Feng Y, Fontenot JD, Rudensky AY (2016). An essential role for the IL-2 receptor in Treg cell function. Nat Immunol.

[118] Shi H, Liu C, Tan H, Li Y, Nguyen TM, Dhungana Y, Guy C, Vogel P, Neale G, Rankin S (2018). Hippo kinases Mst1 and Mst2 sense and amplify IL-2R-STAT5 signaling in regulatory T cells to establish stable regulatory activity. Immunity.

[119] Fleskens V, Minutti CM, Wu X, Wei P, Pals C, McCrae J, Hemmers S, Groenewold V, Vos HJ, Rudensky A (2019). Nemo-like kinase drives Foxp3 stability and is critical for maintenance of immune tolerance by regulatory T cells. Cell Rep.

[120] Chang JH, Hu H, Sun SC (2015). Survival and maintenance of regulatory T cells require the kinase TAK1. Cell Mol Immunol.

[121] Ni X, Kou W, Gu J, Wei P, Wu X, Peng H, Tao J, Yan W, Yang X, Lebid A, et al. TRAF6 directs FOXP3 localization and facilitates regulatory T-cell function through K63-linked ubiquitination. EMBO J. 2019;38(9):e99766. 10.15252/embj.201899766 PMC6484404 30886050

[122] Wen YH, Lin HQ, Li H, Zhao Y, Lui VWY, Chen L, Wu XM, Sun W, Wen WP (2019). Stromal interleukin-33 promotes regulatory T cell-mediated immunosuppression in head and neck squamous cell carcinoma and correlates with poor prognosis. Cancer Immunol Immunother.

[123] Yu F, Sharma S, Edwards J, Feigenbaum L, Zhu J (2015). Dynamic expression of transcription factors T-bet and GATA-3 by regulatory T cells maintains immunotolerance. Nat Immunol.

[124] Li A, Herbst RH, Canner D, Schenkel JM, Smith OC, Kim JY, Hillman M, Bhutkar A, Cuoco MS, Rappazzo CG (2019). IL-33 signaling alters regulatory T cell diversity in support of tumor development. Cell Rep.

[125] Hatzioannou A, Banos A, Sakelaropoulos T, Fedonidis C, Vidali MS, Kohne M, Handler K, Boon L, Henriques A, Koliaraki V (2020). An intrinsic role of IL-33 in Treg cell-mediated tumor immunoevasion. Nat Immunol.

[126] Pastille E, Wasmer MH, Adamczyk A, Vu VP, Mager LF, Phuong NNT, Palmieri V, Simillion C, Hansen W, Kasper S (2019). The IL-33/ST2 pathway shapes the regulatory T cell phenotype to promote intestinal cancer. Mucosal Immunol.

[127] Salem M, Wallace C, Velegraki M, Li A, Ansa-Addo E, Metelli A, Kwon H, Riesenberg B, Wu B, Zhang Y (2019). GARP dampens Cancer immunity by sustaining function and accumulation of regulatory T cells in the Colon. Cancer Res.

[128] Lienart S, Merceron R, Vanderaa C, Lambert F, Colau D, Stockis J, van der Woning B, De Haard H, Saunders M, Coulie PG (2018). Structural basis of latent TGF-beta1 presentation and activation by GARP on human regulatory T cells. Science.

[129] Klann JE, Kim SH, Remedios KA, He Z, Metz PJ, Lopez J, Tysl T, Olvera JG, Ablack JN, Cantor JM (2018). Integrin activation controls regulatory T cell-mediated peripheral tolerance. J Immunol.

[130] Worthington JJ, Kelly A, Smedley C, Bauche D, Campbell S, Marie JC, Travis MA (2015). Integrin alphavbeta8-mediated TGF-beta activation by effector regulatory T cells is essential for suppression of T-cell-mediated inflammation. Immunity.

[131] Sumida T, Lincoln MR, Ukeje CM, Rodriguez DM, Akazawa H, Noda T, Naito AT, Komuro I, Dominguez-Villar M, Hafler DA (2018). Activated beta-catenin in Foxp3(+) regulatory T cells links inflammatory environments to autoimmunity. Nat Immunol.

[132] Baens M, Stirparo R, Lampi Y, Verbeke D, Vandepoel R, Cools J, Marynen P, de Bock CE, Bornschein S (2018). Malt1 self-cleavage is critical for regulatory T cell homeostasis and anti-tumor immunity in mice. Eur J Immunol.

[133] Rosenbaum M, Gewies A, Pechloff K, Heuser C, Engleitner T, Gehring T, Hartjes L, Krebs S, Krappmann D, Kriegsmann M (2019). Bcl10-controlled Malt1 paracaspase activity is key for the immune suppressive function of regulatory T cells. Nat Commun.

[134] Drashansky TT, Helm E, Huo Z, Curkovic N, Kumar P, Luo X, Parthasarathy U, Zuniga A, Cho JJ, Lorentsen KJ (2019). Bcl11b prevents fatal autoimmunity by promoting Treg cell program and constraining innate lineages in Treg cells. Sci Adv.

[135] Hasan SN, Sharma A, Ghosh S, Hong SW, Roy-Chowdhuri S, Im SH, Kang K, Rudra D (2019). Bcl11b prevents catastrophic autoimmunity by controlling multiple aspects of a regulatory T cell gene expression program. Sci Adv.

[136] Ahmetlic F, Riedel T, Homberg N, Bauer V, Trautwein N, Geishauser A, Sparwasser T, Stevanovic S, Rocken M, Mocikat R (2019). Regulatory T cells in an endogenous mouse lymphoma recognize specific antigen peptides and contribute to immune escape. Cancer Immunol Res.

[137] Colbeck EJ, Jones E, Hindley JP, Smart K, Schulz R, Browne M, Cutting S, Williams A, Parry L, Godkin A (2017). Treg depletion licenses T cell-driven HEV Neogenesis and promotes tumor destruction. Cancer Immunol Res.

[138] Akeus P, Szeponik L, Ahlmanner F, Sundstrom P, Alsen S, Gustavsson B, Sparwasser T, Raghavan S, Quiding-Jarbrink M (2018). Regulatory T cells control endothelial chemokine production and migration of T cells into intestinal tumors of APC (min/+) mice. Cancer Immunol Immunother.

[139] Geis AL, Fan H, Wu X, Wu S, Huso DL, Wolfe JL, Sears CL, Pardoll DM, Housseau F (2015). Regulatory T-cell response to Enterotoxigenic Bacteroides fragilis colonization triggers IL17-dependent Colon carcinogenesis. Cancer Discov.

[140] Zhang Y, Lazarus J, Steele NG, Yan W, Lee HJ, Nwosu ZC, Halbrook CJ, Menjivar RE, Kemp SB, Sirihorachai VR (2020). Regulatory T-cell depletion alters the tumor microenvironment and accelerates pancreatic carcinogenesis. Cancer Discov.

[141] Onda M, Kobayashi K, Pastan I (2019). Depletion of regulatory T cells in tumors with an anti-CD25 immunotoxin induces CD8 T cell-mediated systemic antitumor immunity. Proc Natl Acad Sci U S A.

[142] Long Y, Tao H, Karachi A, Grippin AJ, Jin L, Chang YE, Zhang W, Dyson KA, Hou AY, Na M (2020). Dysregulation of glutamate transport enhances Treg function that promotes VEGF blockade resistance in Glioblastoma. Cancer Res.

[143] Kugel CH, Douglass SM, Webster MR, Kaur A, Liu Q, Yin X, Weiss SA, Darvishian F, Al-Rohil RN, Ndoye A (2018). Age correlates with response to anti-PD1, reflecting age-related differences in Intratumoral effector and regulatory T-cell populations. Clin Cancer Res.

[144] Sledzinska A, Vila de Mucha M, Bergerhoff K, Hotblack A, Demane DF, Ghorani E, Akarca AU, MAV M, Solomon I, Vargas FA (2020). Regulatory T cells restrain Interleukin-2- and Blimp-1-dependent Acquisition of Cytotoxic Function by CD4(+) T cells. Immunity.

[145] Kawano Y, Zavidij O, Park J, Moschetta M, Kokubun K, Mouhieddine TH, Manier S, Mishima Y, Murakami N, Bustoros M (2018). Blocking IFNAR1 inhibits multiple myeloma-driven Treg expansion and immunosuppression. J Clin Invest.

[146] Kim BS, Clinton J, Wang Q, Chang SH (2020). Targeting ST2 expressing activated regulatory T cells in Kras-mutant lung cancer. Oncoimmunology.

[147] Tanaka A, Nishikawa H, Noguchi S, Sugiyama D, Morikawa H, Takeuchi Y, Ha D, Shigeta N, Kitawaki T, Maeda Y, et al. Tyrosine kinase inhibitor imatinib augments tumor immunity by depleting effector regulatory T cells. J Exp Med. 2020;217(2):e20191009. 10.1084/jem.20191009 PMC7041710 31704808

[148] Sarhan D, Leijonhufvud C, Murray S, Witt K, Seitz C, Wallerius M, Xie H, Ullen A, Harmenberg U, Lidbrink E (2017). Zoledronic acid inhibits NFAT and IL-2 signaling pathways in regulatory T cells and diminishes their suppressive function in patients with metastatic cancer. Oncoimmunology.

[149] Arce Vargas F, Furness AJS, Solomon I, Joshi K, Mekkaoui L, Lesko MH, Miranda Rota E, Dahan R, Georgiou A, Sledzinska A (2017). Fc-optimized anti-CD25 depletes tumor-infiltrating regulatory T cells and synergizes with PD-1 blockade to eradicate established tumors. Immunity.

[150] Vazquez-Lombardi R, Loetsch C, Zinkl D, Jackson J, Schofield P, Deenick EK, King C, Phan TG, Webster KE, Sprent J, Christ D (2017). Potent antitumour activity of interleukin-2-fc fusion proteins requires fc-mediated depletion of regulatory T-cells. Nat Commun.

[151] Shabaneh TB, Molodtsov AK, Steinberg SM, Zhang P, Torres GM, Mohamed GA, Boni A, Curiel TJ, Angeles CV, Turk MJ (2018). Oncogenic BRAF(V600E) governs regulatory T-cell recruitment during melanoma tumorigenesis. Cancer Res.

[152] Ni X, Jorgensen JL, Goswami M, Challagundla P, Decker WK, Kim YH, Duvic MA (2015). Reduction of regulatory T cells by Mogamulizumab, a defucosylated anti-CC chemokine receptor 4 antibody, in patients with aggressive/refractory mycosis fungoides and Sezary syndrome. Clin Cancer Res.

[153] Wang R, Feng W, Wang H, Wang L, Yang X, Yang F, Zhang Y, Liu X, Zhang D, Ren Q (2020). Blocking migration of regulatory T cells to leukemic hematopoietic microenvironment delays disease progression in mouse leukemia model. Cancer Lett.

[154] Wang D, Yang L, Yu W, Wu Q, Lian J, Li F, Liu S, Li A, He Z, Liu J (2019). Colorectal cancer cell-derived CCL20 recruits regulatory T cells to promote chemoresistance via FOXO1/CEBPB/NF-kappaB signaling. J Immunother Cancer.

[155] Luo CT, Liao W, Dadi S, Toure A, Li MO (2016). Graded Foxo1 activity in Treg cells differentiates tumour immunity from spontaneous autoimmunity. Nature.

[156] Wang X, Lang M, Zhao T, Feng X, Zheng C, Huang C, Hao J, Dong J, Luo L, Li X (2017). Cancer-FOXP3 directly activated CCL5 to recruit FOXP3(+)Treg cells in pancreatic ductal adenocarcinoma. Oncogene.

[157] Jackson JJ, Ketcham JM, Younai A, Abraham B, Biannic B, Beck HP, Bui MHT, Chian D, Cutler G, Diokno R (2019). Discovery of a potent and selective CCR4 antagonist that inhibits Treg trafficking into the tumor microenvironment. J Med Chem.

[158] Maeda S, Murakami K, Inoue A, Yonezawa T, Matsuki N (2019). CCR4 blockade depletes regulatory T cells and prolongs survival in a canine model of bladder Cancer. Cancer Immunol Res.

[159] Hirata A, Hashimoto H, Shibasaki C, Narumi K, Aoki K (2019). Intratumoral IFN-alpha gene delivery reduces tumor-infiltrating regulatory T cells through the downregulation of tumor CCL17 expression. Cancer Gene Ther.

[160] Rapp M, Wintergerst MWM, Kunz WG, Vetter VK, Knott MML, Lisowski D, Haubner S, Moder S, Thaler R, Eiber S (2019). CCL22 controls immunity by promoting regulatory T cell communication with dendritic cells in lymph nodes. J Exp Med.

[161] Wiedemann GM, Knott MM, Vetter VK, Rapp M, Haubner S, Fesseler J, Kuhnemuth B, Layritz P, Thaler R, Kruger S (2016). Cancer cell-derived IL-1alpha induces CCL22 and the recruitment of regulatory T cells. Oncoimmunology.

[162] Plitas G, Konopacki C, Wu K, Bos PD, Morrow M, Putintseva EV, Chudakov DM, Rudensky AY (2016). Regulatory T cells exhibit distinct features in human breast Cancer. Immunity.

[163] Kim HR, Park HJ, Son J, Lee JG, Chung KY, Cho NH, Shim HS, Park S, Kim G, In Yoon H (2019). Tumor microenvironment dictates regulatory T cell phenotype: Upregulated immune checkpoints reinforce suppressive function. J Immunother Cancer.

[164] Liu Y, Zheng P (2020). Preserving the CTLA-4 checkpoint for safer and more effective Cancer immunotherapy. Trends Pharmacol Sci.

[165] Sharma A, Subudhi SK, Blando J, Scutti J, Vence L, Wargo J, Allison JP, Ribas A, Sharma P (2019). Anti-CTLA-4 immunotherapy does not deplete FOXP3(+) regulatory T cells (Tregs) in human cancers. Clin Cancer Res.

[166] Arce Vargas F, Furness AJS, Litchfield K, Joshi K, Rosenthal R, Ghorani E, Solomon I, Lesko MH, Ruef N, Roddie C (2018). Fc effector function contributes to the activity of human anti-CTLA-4 antibodies. Cancer Cell.

[167] Ingram JR, Blomberg OS, Rashidian M, Ali L, Garforth S, Fedorov E, Fedorov AA, Bonanno JB, Le Gall C, Crowley S (2018). Anti-CTLA-4 therapy requires an fc domain for efficacy. Proc Natl Acad Sci U S A.

[168] Du X, Tang F, Liu M, Su J, Zhang Y, Wu W, Devenport M, Lazarski CA, Zhang P, Wang X (2018). A reappraisal of CTLA-4 checkpoint blockade in cancer immunotherapy. Cell Res.

[169] Sanseviero E, O'Brien EM, Karras JR, Shabaneh TB, Aksoy BA, Xu W, Zheng C, Yin X, Xu X, Karakousis GC (2019). Anti-CTLA-4 activates Intratumoral NK cells and combined with IL15/IL15Ralpha complexes enhances tumor control. Cancer Immunol Res.

[170] Cari L, Nocentini G, Migliorati G, Riccardi C (2018). Potential effect of tumor-specific Treg-targeted antibodies in the treatment of human cancers: a bioinformatics analysis. Oncoimmunology.

[171] Ha D, Tanaka A, Kibayashi T, Tanemura A, Sugiyama D, Wing JB, Lim EL, Teng KWW, Adeegbe D, Newell EW (2019). Differential control of human Treg and effector T cells in tumor immunity by fc-engineered anti-CTLA-4 antibody. Proc Natl Acad Sci U S A.

[172] Ren ZH, Fu YX (2019). Degradation of CTLA-4 balances toxicity and efficacy. Science Bulletin.

[173] Larkin J, Chiarion-Sileni V, Gonzalez R, Grob JJ, Cowey CL, Lao CD, Schadendorf D, Dummer R, Smylie M, Rutkowski P (2015). Combined Nivolumab and Ipilimumab or Monotherapy in untreated melanoma. N Engl J Med.

[174] Du X, Liu M, Su J, Zhang P, Tang F, Ye P, Devenport M, Wang X, Zhang Y, Liu Y, Zheng P (2018). Uncoupling therapeutic from immunotherapy-related adverse effects for safer and effective anti-CTLA-4 antibodies in CTLA4 humanized mice. Cell Res.

[175] Zhang Y, Du X, Liu M, Tang F, Zhang P, Ai C, Fields JK, Sundberg EJ, Latinovic OS, Devenport M (2019). Hijacking antibody-induced CTLA-4 lysosomal degradation for safer and more effective cancer immunotherapy. Cell Res.

[176] Sharma N, Vacher J, Allison JP (2019). TLR1/2 ligand enhances antitumor efficacy of CTLA-4 blockade by increasing intratumoral Treg depletion. Proc Natl Acad Sci U S A.

[177] Dorta-Estremera S, Hegde VL, Slay RB, Sun R, Yanamandra AV, Nicholas C, Nookala S, Sierra G, Curran MA, Sastry KJ (2019). Targeting interferon signaling and CTLA-4 enhance the therapeutic efficacy of anti-PD-1 immunotherapy in preclinical model of HPV(+) oral cancer. J Immunother Cancer.

[178] Kvarnhammar AM, Veitonmaki N, Hagerbrand K, Dahlman A, Smith KE, Fritzell S, von Schantz L, Thagesson M, Werchau D, Smedenfors K (2019). The CTLA-4 x OX40 bispecific antibody ATOR-1015 induces anti-tumor effects through tumor-directed immune activation. J Immunother Cancer.

[179] Goswami S, Apostolou I, Zhang J, Skepner J, Anandhan S, Zhang X, Xiong L, Trojer P, Aparicio A, Subudhi SK (2018). Modulation of EZH2 expression in T cells improves efficacy of anti-CTLA-4 therapy. J Clin Invest.

[180] Coutzac C, Jouniaux JM, Paci A, Schmidt J, Mallardo D, Seck A, Asvatourian V, Cassard L, Saulnier P, Lacroix L (2020). Systemic short chain fatty acids limit antitumor effect of CTLA-4 blockade in hosts with cancer. Nat Commun.

[181] Kurtulus S, Sakuishi K, Ngiow SF, Joller N, Tan DJ, Teng MW, Smyth MJ, Kuchroo VK, Anderson AC (2015). TIGIT predominantly regulates the immune response via regulatory T cells. J Clin Invest.

[182] Shen YC, Ghasemzadeh A, Kochel CM, Nirschl TR, Francica BJ, Lopez-Bujanda ZA, Carrera Haro MA, Tam A, Anders RA, Selby MJ (2018). Combining intratumoral Treg depletion with androgen deprivation therapy (ADT): preclinical activity in the Myc-CaP model. Prostate Cancer Prostatic Dis.

[183] Langhans B, Nischalke HD, Kramer B, Dold L, Lutz P, Mohr R, Vogt A, Toma M, Eis-Hubinger AM, Nattermann J (2019). Role of regulatory T cells and checkpoint inhibition in hepatocellular carcinoma. Cancer Immunol Immunother.

[184] Kamada T, Togashi Y, Tay C, Ha D, Sasaki A, Nakamura Y, Sato E, Fukuoka S, Tada Y, Tanaka A (2019). PD-1(+) regulatory T cells amplified by PD-1 blockade promote hyperprogression of cancer. Proc Natl Acad Sci U S A.

[185] Mahne AE, Mauze S, Joyce-Shaikh B, Xia J, Bowman EP, Beebe AM, Cua DJ, Jain R (2017). Dual roles for regulatory T-cell depletion and Costimulatory signaling in agonistic GITR targeting for tumor immunotherapy. Cancer Res.

[186] Woods DM, Ramakrishnan R, Laino AS, Berglund A, Walton K, Betts BC, Weber JS (2018). Decreased suppression and increased phosphorylated STAT3 in regulatory T cells are associated with benefit from adjuvant PD-1 blockade in resected metastatic melanoma. Clin Cancer Res.

[187] Hossain DM, Panda AK, Manna A, Mohanty S, Bhattacharjee P, Bhattacharyya S, Saha T, Chakraborty S, Kar RK, Das T (2013). FoxP3 acts as a cotranscription factor with STAT3 in tumor-induced regulatory T cells. Immunity.

[188] Oweida AJ, Darragh L, Phan A, Binder D, Bhatia S, Mueller A, Court BV, Milner D, Raben D, Woessner R (2019). STAT3 modulation of regulatory T cells in response to radiation therapy in head and neck Cancer. J Natl Cancer Inst.

[189] DiDomenico J, Lamano JB, Oyon D, Li Y, Veliceasa D, Kaur G, Ampie L, Choy W, Lamano JB, Bloch O (2018). The immune checkpoint protein PD-L1 induces and maintains regulatory T cells in glioblastoma. Oncoimmunology.

[190] Taylor NA, Vick SC, Iglesia MD, Brickey WJ, Midkiff BR, McKinnon KP, Reisdorf S, Anders CK, Carey LA, Parker JS (2017). Treg depletion potentiates checkpoint inhibition in claudin-low breast cancer. J Clin Invest.

[191] Liu Z, McMichael EL, Shayan G, Li J, Chen K, Srivastava R, Kane LP, Lu B, Ferris RL (2018). Novel effector phenotype of Tim-3(+) regulatory T cells leads to enhanced suppressive function in head and neck Cancer patients. Clin Cancer Res.

[192] Oweida A, Hararah MK, Phan A, Binder D, Bhatia S, Lennon S, Bukkapatnam S, Van Court B, Uyanga N, Darragh L (2018). Resistance to radiotherapy and PD-L1 blockade is mediated by TIM-3 Upregulation and regulatory T-cell infiltration. Clin Cancer Res.

[193] Liu JF, Wu L, Yang LL, Deng WW, Mao L, Wu H, Zhang WF, Sun ZJ (2018). Blockade of TIM3 relieves immunosuppression through reducing regulatory T cells in head and neck cancer. J Exp Clin Cancer Res.

[194] Le KS, Thibult ML, Just-Landi S, Pastor S, Gondois-Rey F, Granjeaud S, Broussais F, Bouabdallah R, Colisson R, Caux C (2016). Follicular B lymphomas generate regulatory T cells via the ICOS/ICOSL pathway and are susceptible to treatment by anti-ICOS/ICOSL therapy. Cancer Res.

[195] Sim GC, Liu C, Wang E, Liu H, Creasy C, Dai Z, Overwijk WW, Roszik J, Marincola F, Hwu P (2016). IL2 variant circumvents ICOS+ regulatory T-cell expansion and promotes NK cell activation. Cancer Immunol Res.

[196] Kachler K, Holzinger C, Trufa DI, Sirbu H, Finotto S (2018). The role of Foxp3 and Tbet co-expressing Treg cells in lung carcinoma. Oncoimmunology.

[197] Arterbery AS, Osafo-Addo A, Avitzur Y, Ciarleglio M, Deng Y, Lobritto SJ, Martinez M, Hafler DA, Kleinewietfeld M, Ekong UD (2016). Production of Proinflammatory cytokines by monocytes in liver-transplanted recipients with De novo autoimmune hepatitis is enhanced and induces TH1-like regulatory T cells. J Immunol.

[198] Kitz A, de Marcken M, Gautron AS, Mitrovic M, Hafler DA, Dominguez-Villar M (2016). AKT isoforms modulate Th1-like Treg generation and function in human autoimmune disease. EMBO Rep.

[199] Ouyang W, Liao W, Luo CT, Yin N, Huse M, Kim MV, Peng M, Chan P, Ma Q, Mo Y (2012). Novel Foxo1-dependent transcriptional programs control T (reg) cell function. Nature.

[200] Ouyang W, Beckett O, Ma Q, Paik JH, DePinho RA, Li MO (2010). Foxo proteins cooperatively control the differentiation of Foxp3+ regulatory T cells. Nat Immunol.

[201] Lee JH, Elly C, Park Y, Liu YC (2015). E3 ubiquitin ligase VHL regulates hypoxia-inducible factor-1alpha to maintain regulatory T cell stability and suppressive capacity. Immunity.

[202] Jung K, Kim JA, Kim YJ, Lee HW, Kim CH, Haam S, Kim YS (2020). A Neuropilin-1 antagonist exerts antitumor immunity by inhibiting the suppressive function of Intratumoral regulatory T cells. Cancer Immunol Res.

[203] Liu Y, Zhang L, Wang B, Yang Z, Xu G, Ma A, Tang M, Jing T, Wu L, Xu X, Liu Y (2019). Requirement for POH1 in differentiation and maintenance of regulatory T cells. Cell Death Differ.

[204] Chellappa S, Kushekhar K, Munthe LA, Tjonnfjord GE, Aandahl EM, Okkenhaug K, Tasken K (2019). The PI3K p110delta isoform inhibitor Idelalisib preferentially inhibits human regulatory T cell function. J Immunol.

[205] Ali K, Soond DR, Pineiro R, Hagemann T, Pearce W, Lim EL, Bouabe H, Scudamore CL, Hancox T, Maecker H (2014). Inactivation of PI(3) K p110delta breaks regulatory T-cell-mediated immune tolerance to cancer. Nature.

