# Supplementary material for: Regulatory T cells in tumor microenvironment: new mechanisms, potential therapeutic strategies and future prospects
Source: Mol Cancer. 2020 Jul 17;19:116. doi: 10.1186/s12943-020-01234-1 (PMC7367382; doi:10.1186/s12943-020-01234-1)
Supplement: Supplementary file 1 — Additional file 1. [file 12943_2020_1234_MOESM1_ESM.docx]

**Supplementary Table 1. Clinical trials targeting regulatory T cells**

| **Rank** | **Title** | **Status** | **NCT** |
| --- | --- | --- | --- |
| 1 | The Effect of Chemotherapy on Regulatory T Cells | Unknown status | NCT03413046 |
| 2 | Allogeneic Immunotherapy for Hematological Malignancies by Selective Depletion of Regulatory T Cells | Not yet recruiting | NCT03236129 |
| 3 | Regulatory T-cells in Psoriasis Patients as Targets for Therapy | Completed | NCT01233583 |
| 4 | Regulatory T-cells and Crohn's Disease | Completed | NCT02060318 |
| 5 | Characterization of Regulatory T Lymphocytes in the Synovial Fluid of Patients Affected by Rheumatoid Arthritis. | Recruiting | NCT03293667 |
| 6 | Safety and Efficacy Study of Regulatory T Cells in Treating Autoimmune Hepatitis | Unknown status | NCT02704338 |
| 7 | A Phase 1/2 Trial of Donor Regulatory T-cells for Steroid-Refractory Chronic Graft-versus-Host-Disease | Unknown status | NCT02385019 |
| 8 | Polyclonal Regulatory T Cells (PolyTregs) for Pemphigus | Recruiting | NCT03239470 |
| 9 | Regulatory T Cells in COPD | Completed | NCT00452764 |
| 10 | Regulatory T Cells (Tregs) in Polymorphic Light Eruption | Completed | NCT00555178 |
| 11 | Function of Regulatory T Cells Improved by Dexamethasone in Graves' Patients | Completed | NCT01534169 |
| 12 | Regulatory T Cells in Type 1 Diabetes Patients Treated With IL-2 | Completed | NCT01827735 |
| 13 | Safety and Efficacy of Umbilical Cord Blood Regulatory T Cells Plus Liraglutide on Autoimmune Diabetes | Recruiting | NCT03011021 |
| 14 | Rapamycin and Regulatory T Cells in Kidney Transplantation | Completed | NCT01014234 |
| 15 | Adaptive Study of IL-2 Dose Frequency on Regulatory T Cells in Type 1 Diabetes | Completed | NCT02265809 |
| 16 | Induction of Regulatory t Cells by Low Dose il2 in Autoimmune and Inflammatory Diseases | Recruiting | NCT01988506 |
| 17 | Effects of High Cut-off (HCO) Hemodialysis on Central Memory CD4+ T and Treg Cells in Patients With End-stage Kidney Disease | Unknown status | NCT01103076 |
| 18 | Donor Regulatory T Cells Infusion in Patients With Chronic Graft-versus-host Disease (GVHD) | Recruiting | NCT01903473 |
| 19 | Role of Regulatory T Cells in Pathogenesis of Primary IgA Nephropathy | Completed | NCT00521508 |
| 20 | Effect of Different Therapeutic Strategies on Regulatory T Cells in Kidney Transplantation | Completed | NCT01640743 |
| 21 | Safety Study of Using Regulatory T Cells Induce Liver Transplantation Tolerance | Unknown status | NCT01624077 |
| 22 | Donor Regulatory T-cells for Steroid-Refractory Chronic Graft-versus-host-Disease | Recruiting | NCT03683498 |
| 23 | Infusion of Donor Lymphocytes Depleted of CD25+ Regulatory T-cells in Patients With Relapsed Hematologic Malignancies | Completed | NCT00675831 |
| 24 | Use of Erythropoietin to Expand Regulatory T Cells in Autoimmune Liver Disease | Enrolling by invitation | NCT03842254 |
| 25 | Trial of Regulatory T-cells Plus Low-Dose Interleukin-2 for Steroid-Refractory Chronic Graft-versus-Host-Disease | Active, not recruiting | NCT01937468 |
| 26 | Amplifying Graft-Versus-Tumor Effect by Donor Regulatory T-Cell Depletion Before Donor Lymphocytes Infusion | Completed | NCT00987987 |
| 27 | Low-dose IL-2 to the Kinetics of Regulatory T-cell in Healthy Volunteers | Recruiting | NCT03837093 |
| 28 | Donor-Alloantigen-Reactive Regulatory T Cell (darTreg) Therapy in Renal Transplantation (The ONE Study ) | Completed | NCT02244801 |
| 29 | A Pilot Study Using Autologous Regulatory T Cell Infusion Zortress (Everolimus) in Renal Transplant Recipients | Recruiting | NCT03284242 |
| 30 | Safety and Efficacy Study of Regulatory T Cell Therapy in Liver Transplant Patients | Completed | NCT02166177 |
| 31 | Inducible Regulatory T Cells (iTregs) in Non-Myeloablative Sibling Donor Peripheral Blood Stem Cell Transplantation | Completed | NCT01634217 |
| 32 | Ex-vivo Expanded Donor Regulatory T Cells for Prevention of Acute Graft-Versus-Host Disease | Recruiting | NCT01795573 |
| 33 | Efficacy of Low Dose, SubQ Interleukin-2 (IL-2) to Expand Endogenous Regulatory T-Cells in Liver Transplant Recipients | Active, not recruiting | NCT02739412 |
| 34 | Augmentation of Dendritic Cell-Based Vaccines in Melanoma Patients by Depletion of Regulatory T Cells in Stage IV Melanoma Patients | Completed | NCT00847106 |
| 35 | Safety and Efficacy Study of Co-transfering of Mesenchymal Stem Cell and Regulatory T Cells in Treating End-stage Liver Disease | Not yet recruiting | NCT03460795 |
| 36 | Evaluating the Effect of Isotretinoin in Regulatory T-cell Function in Adverse Cutaneous Drug Eruptions (ACDEs): A Pilot Study | Recruiting | NCT02795143 |
| 37 | Continuous Alloreactive T Cell Depletion and Regulatory T Cell Expansion for the Treatment of Steroid-refractory or Dependent Chronic GVHD | Active, not recruiting | NCT02519816 |
| 38 | The Role of Regulatory T Cell in Ovarian Cancer: Focus on Relationship Between Clinical Prognosis and Regulatory T Cell Expression | Unknown status | NCT00854282 |
| 39 | Role of T Helper 17 and Regulatory T Cells in Delayed Graft Function | Unknown status | NCT01232816 |
| 40 | Elimination of CD4+CD25+ Regulatory T Cells in Patients With Hepatocellular Carcinoma | Completed | NCT00396682 |
| 41 | Phase 1-2 MAHCT w/ TCell Depleted Graft w/ Simultaneous Infusion Conventional and Regulatory T Cell | Recruiting | NCT01660607 |
| 42 | Observation on the Treg in the Uveitis Patients | Completed | NCT01306474 |
| 43 | Multiple Donor Treg DLI for Severe Refractory Chronic GVHD | Recruiting | NCT02749084 |
| 44 | In-vivo Regulatory T Cell Enhancement With Cyclophosphamide and Sirolimus With or Without Vidaza (Azacitidine) for Steroid-refractory Acute Graft-versus-host Disease | Completed | NCT01453140 |
| 45 | Regulatory T-cells After Subcutaneous Immunotherapy | Completed | NCT01830673 |
| 46 | Distinct Response of CD4+CD25+Foxp3+ and IL-10-secreting Type I T Regulatory Cells to Cluster Specific Immunotherapy in Allergic Rhinitis Children | Completed | NCT01291381 |
| 47 | Safety and Immunogenicity of Recombinant WT1 Antigen-Specific Cancer Immunotherapeutic Combined With Infusion of Treg Depleted T Cells for Adult WT1 Acute Myeloid Leukemia | Unknown status | NCT01513109 |
| 48 | Treg Modulation With CD28 and IL-6 Receptor Antagonists | Recruiting | NCT04066114 |
| 49 | Safety Study and Therapeutic Effects of Umbilical Cord Blood Treg on Autoimmune Diabetes | Recruiting | NCT02932826 |
| 50 | T Regulatory Cells in Hepatitis c Infected Patients | Completed | NCT03186235 |
| 51 | Effects of Sevoflurane and Desflurane on Treg | Completed | NCT02559297 |
| 52 | T-Regulatory Cells in Amyotrophic Lateral Sclerosis | Unknown status | NCT03241784 |
| 53 | Safety and Efficacy of Allogeneic MSCs in Promoting T-regulatory Cells in Patients With Small Abdominal Aortic Aneurysms | Recruiting | NCT02846883 |
| 54 | Treg Adoptive Therapy for Subclinical Inflammation in Kidney Transplantation | Unknown status | NCT02088931 |
| 55 | Combination With Treg Levels and CMR to Assess the Severity and Prognosis of Reperfusion Injury After PPCI in STEMI Patients | Recruiting | NCT03939338 |
| 56 | T Regulatory Lymphocytes (Treg) Depletion for Cancer Treatment Efficacy and Safety Study | Completed | NCT00986518 |
| 57 | T1DM Immunotherapy Using CD4+CD127lo/-CD25+ Polyclonal Tregs | Completed | NCT01210664 |
| 58 | Liver Transplantation With Tregs at MGH | Recruiting | NCT03577431 |
| 59 | Response to Tregs in Innate Immunity Receptor LRP1 (CD91) and Tregs in Periferic Blood Mononuclear Cells in Patients With Non-segmentary Vitiligo | Completed | NCT03249064 |
| 60 | T1DM Immunotherapy Using Polyclonal Tregs + IL-2 | Active, not recruiting | NCT02772679 |
| 61 | Treg Therapy in Subclinical Inflammation in Kidney Transplantation | Recruiting | NCT02711826 |
| 62 | The Immune Tolerance Mechanism Induced by IL-17-producing Regulatory T Cells in the Orthotopic Liver Transplant Recipients With Aspergillosis | Unknown status | NCT01117077 |
| 63 | Association of Breg and Treg With the Clinical Effects of Infliximab in the Treatment of Patients With Crohn's Disease | Completed | NCT04272788 |
| 64 | TLI, ATG & Hematopoietic Stem Cell Transplantation and Recipient T Regs Therapy in Living Donor Kidney Transplantation | Not yet recruiting | NCT03943238 |
| 65 | Nivolumab in mRCC Patients: Treg Function, T-cell Access and NK Interactions to Predict and Improve Efficacy | Recruiting | NCT03891485 |
| 66 | Study of Phenotypic and Functional Characteristics of Regulatory T Lymphocytes in Horton's Disease | Completed | NCT02857192 |
| 67 | Donor Alloantigen Reactive Tregs (darTregs) for Calcineurin Inhibitor (CNI) Reduction | Completed | NCT02474199 |
| 68 | Effects of Montelukast on Airway Regulatory T Cells in Asthma | Unknown status | NCT01951898 |
| 69 | Maternal Plasmatic Regulatory T Cells and Th17 as Possible Diagnosis Markers of Acute Chorioamnionitis | Unknown status | NCT01610258 |
| 70 | The Changes of Treg Cells Frequency and Function During Antiviral Therapy | Unknown status | NCT03210493 |
| 71 | T-regulatory Cells in ALS | Active, not recruiting | NCT04055623 |
| 72 | Study of T-Regulatory Cells in Asthma | Completed | NCT02574351 |
| 73 | T-regulatory Cells in Diabetic Type Two Nephropathy | Not yet recruiting | NCT03591939 |
| 74 | Intrauterine Human Chorionic Gonadotrophins (hCG) and Endometrial Treg Cells | Completed | NCT01064219 |
| 75 | T Regulatory Cells in Hemodialysis Patients: Observational Study | Completed | NCT02981992 |
| 76 | T Regulatory Cells in Renal Cell Carcinoma (PILOT STUDY) | Completed | NCT00717743 |
| 77 | Treg Immunotherapy in Crohn's Disease | Not yet recruiting | NCT03185000 |
| 78 | Immunoregulatory Mechanisms of Treg Cells Induced by Tocilizumab | Completed | NCT02963402 |
| 79 | TREg Activation in the Treatment of the PELADE (Alopecia Areata) | Completed | NCT02557074 |
| 80 | Different Doses of Vitamin D and T Regulatory Cells in Preterm Infants | Completed | NCT03793309 |
| 81 | Infusion of T-Regulatory Cells in Kidney Transplant Recipients (The ONE Study) | Unknown status | NCT02091232 |
| 82 | In Vivo Treg Expansion and Graft-Versus-Host Disease Prophylaxis | Completed | NCT01927120 |
| 83 | The Th17/Treg Cells and IL-23/IL-17 Axis and Early Enteral Nutrition in Sepsis | Completed | NCT03385850 |
| 84 | A Phase 1 Trial of CD25/Treg-depleted DLI Plus Ipilimumab for Myeloid Disease Relapse After Matched-HCT | Recruiting | NCT03912064 |
| 85 | The Role of Sodium Chloride and the Treg/Th17 Axis in Autoimmune Hepatitis | Recruiting | NCT02050646 |
| 86 | Low-dose Interleukin-2 in Women With Unexplained Miscarriages | Not yet recruiting | NCT03970954 |
| 87 | Phase 1 Infused Donor T Regulatory Cells in Steroid Dependent/Refractory Chronic GVHD | Unknown status | NCT01911039 |
| 88 | The Effects of Nanocurcumin on Treg Cells and Th17 Cells Responses in Ankylosing Spondylitis Patients | Completed | NCT03140657 |
| 89 | Potentiation of Cetuximab by Tregs Depletion With CSA in Advanced Head & Neck Cancer | Completed | NCT01581970 |
| 90 | T-Regulatory Cell Infusion Post Umbilical Cord Blood Transplant in Patients With Advanced Hematologic Cancer | Completed | NCT00602693 |
| 91 | Enumeration and Function Analysis of Treg Cells in Peripheral Blood of HCC Patients Before and After Ablation Therapy | Unknown status | NCT01668381 |
| 92 | Fucosylated T Cells for Graft Versus Host Disease (GVHD) Prevention | Active, not recruiting | NCT02423915 |
| 93 | Dose Finding Study of Il-2 at Ultra-low Dose in Children With Recently Diagnosed Type 1 Diabetes | Unknown status | NCT01862120 |
| 94 | The Role of Regulatory T Cell in Patients With Type 1 Diabetes Mellitus | Unknown status | NCT00173641 |
| 95 | Safety and Efficacy of CLBS03 in Adolescents With Recent Onset Type 1 Diabetes (The Sanford Project T-Rex Study) | Active, not recruiting | NCT02691247 |
| 96 | PolyTreg Immunotherapy in Islet Transplantation | Recruiting | NCT03444064 |
| 97 | Basiliximab in Treating Patients With Newly Diagnosed Glioblastoma Multiforme Undergoing Targeted Immunotherapy and Temozolomide-Caused Lymphopenia | Completed | NCT00626483 |
| 98 | Cell Therapy for Immunomodulation in Kidney Transplantation | Recruiting | NCT03867617 |
| 99 | Ultra-low Dose Subcutaneous IL-2 in Renal Transplantation | Unknown status | NCT02417870 |
| 100 | Effect of Synbiotic on Immune Response, Gut Permeability and Microbiota in Patient With Connective Tissue Disease | Completed | NCT03494036 |
| 101 | Low Dose IL-2, Hematopoietic Stem Cell Transplantation, IL2 for GVHD | Completed | NCT00539695 |
| 102 | A Study of Engineered Donor Grafts (TregGraft) in Recipients Undergoing Allogeneic Transplantation for Hematologic Malignancies | Recruiting | NCT04013685 |
| 103 | A Randomized Prospective Clinical Trial of Fel d 1 Peptide Immunotherapy | Completed | NCT02311413 |
| 104 | Dose-effect Relationship of Low-dose IL-2 in Type 1 Diabetes | Completed | NCT01353833 |
| 105 | Studies of Immune Responses in Patients With Chronic Hepatitis B | Completed | NCT00155155 |
| 106 | Vaccination Plus Ontak in Patients With Metastatic Melanoma | Unknown status | NCT00515528 |
| 107 | S8809-S9800-S9911TM- Biological Markers in Patients With Follicular Lymphoma Treated on Clinical Trial SWOG-8809, SWOG-9800, or SWOG-9911 | Completed | NCT00896922 |
| 108 | Denileukin Diftitox Followed by Vaccine Therapy in Treating Patients With Metastatic Cancer | Completed | NCT00128622 |
| 109 | Leuven Tolerogenic Protocol for Intestinal Transplantation | Unknown status | NCT02314949 |
| 110 | RAPA-501 Therapy for ALS | Not yet recruiting | NCT04220190 |
| 111 | Treatment of Children With Kidney Transplants by Injection of CD4+CD25+FoxP3+ T Cells to Prevent Organ Rejection | Unknown status | NCT01446484 |
| 112 | The Role of the Thymus in Type I Diabetes. | Unknown status | NCT03236558 |
| 113 | Treg, Th17 Cells, NKT in Epithelial Ovarian Tumor | Completed | NCT03779399 |
| 114 | The ONE Study UK Treg Trial | Completed | NCT02129881 |
| 115 | Irradiation-based Myeloablative Conditioning Followed by Treg/Tcon Immunotherapy in HSCT | Recruiting | NCT03977103 |
| 116 | The Immunomodulatory Effects of Oral Nanocurcumin in Multiple Sclerosis Patients | Completed | NCT03150966 |
| 117 | Trial of Adoptive Immunotherapy With TRACT to Prevent Rejection in Living Donor Kidney Transplant Recipients | Completed | NCT02145325 |
| 118 | Characterizing Biomarkers of Early Parkinson's Disease Progression (TREG) | Recruiting | NCT03716258 |
| 119 | Children With HIV and Asthma (CHIVAS) | Completed | NCT01644370 |
| 120 | Effect of Denileukin Diftitox on Immune System in CTCL Patients | Completed | NCT00254332 |
| 121 | Lactoferrin Prophylaxis in VLBW and Regulator T-cells | Completed | NCT01287507 |
| 122 | Gut Priming With Oral Bovine Colostrum for Preterm Neonates; Randomized Control Trial | Completed | NCT03926390 |
| 123 | Regulatory Lymphocytes in Patients Treated With Specific Immunotherapy | Completed | NCT01475188 |
| 124 | The ONE Study nTreg Trial (ONEnTreg13) | Completed | NCT02371434 |
| 125 | Vaccine Therapy and Basiliximab in Treating Patients With Acute Myeloid Leukemia in Complete Remission | Completed | NCT01842139 |
| 126 | Effect of Prednisone onTregs and TH17 | Completed | NCT01002313 |
| 127 | A Study to Evaluate the Safety and Tolerability of RO7296682 in Participants With Advanced Solid Tumors. | Recruiting | NCT04158583 |
| 128 | Efficacy of Acupuncture and Moxibustion Treatment in Patients With Active Crohn's Disease | Completed | NCT01697761 |
| 129 | MIROCALS: Modifying Immune Response and OutComes in ALS | Active, not recruiting | NCT03039673 |
| 130 | Celecoxib in Treating Patients With Stage IIIB or Stage IV Non-Small Cell Lung Cancer | Completed | NCT00104767 |
| 131 | Circulating Regulatory Lymphocytes and Outcome of Metastatic Colorectal Cancer Patients | Completed | NCT01533740 |
| 132 | The Differential Effects of 3 Different Immunosuppressive | Completed | NCT00729248 |
| 133 | Vitamin D Supplementation and Regulatory FoxP3+ T Cells in the GUT | Completed | NCT01538485 |
| 134 | A Study of NKTR-358 (LY3471851) in Participants With Systemic Lupus Erythematosus (SLE) | Completed | NCT03556007 |
| 135 | Evaluation Effect of Crocina on The Cellular Immune Responses in Osteoarthritis Patients | Unknown status | NCT03375814 |
| 136 | Study of the Role of Regulator T Cells in the Pathophysiology of Childhood Henoch Sch枚nlein Purpura | Completed | NCT02317133 |
| 137 | LOW DOSE IL-2 FOR THE TREATMENT OF CROHN'S DISEASE | Not yet recruiting | NCT04263831 |
| 138 | Efficacy of Rapamycin Therapy With Chronic Immune Thrombocytopenia | Completed | NCT01672151 |
| 139 | Low Dose IL-2 Therapy in Patients With a Depressive Episode in the Course of a Bipolar Disorder | Not yet recruiting | NCT04133233 |
| 140 | Evaluating the Interest of Interleukine-2 for Patients With Active Warm Hemolytic Anemia Resistant to Conventional Treatment | Completed | NCT02389231 |
| 141 | A Safety and Tolerability Study of GB301 | Not yet recruiting | NCT03865017 |
| 142 | The Number and Function of Regular T Cell in Patients With Obstructive Sleep Apnea | Unknown status | NCT02342028 |
| 143 | Evaluation of Corticosteroid in Systemic Inflammatory Response Syndrome | Not yet recruiting | NCT03876041 |
| 144 | Vitamin D Supplementation in Systemic Lupus Erythematosus | Completed | NCT01413230 |
| 145 | The Role of FGL2-FcgammaRIIB Inhibitory Pathway in Human Viral Hepatitis | Completed | NCT01711164 |
| 146 | Biological Activity and Safety of Low Dose IL2 in Relapsing Remitting Multiple Sclerosis | Active, not recruiting | NCT02424396 |
| 147 | Study of ADCT-301 in Patients With Selected Advanced Solid Tumors | Recruiting | NCT03621982 |
| 148 | Safety and Efficacy of Abatacept for Treating Chronic Cytopenia in Cytotoxic T-Lymphocyte Antigen 4 (CTLA4) Haploinsufficiency | Not yet recruiting | NCT03733067 |
| 149 | Vitamin D3 Supplementation and the T Cell Compartment in Multiple Sclerosis (MS) | Completed | NCT00940719 |
| 150 | Ovarian Dendritic Cell Vaccine Trial | Recruiting | NCT00703105 |
| 151 | Decitabine Augments for Post Allogeneic Stem Cell Transplantation in Patients With Acute Myeloid Leukemia and Myelodysplastic Syndrome | Unknown status | NCT01809392 |
| 152 | Immunological Mechanisms of Oralair庐 in Patients With Seasonal Allergic Rhinitis | Unknown status | NCT02014623 |
| 153 | Worms for Immune Regulation of Multiple Sclerosis | Completed | NCT01470521 |
| 154 | Study to Evaluate the Tolerability and Immunogenicity of Nyaditum Resae 庐 Probiotic Administered to Pediatric Population in Contact With Tuberculosis With or Without Latent Tuberculosis Infection | Completed | NCT02581579 |
| 155 | Safety and Immunogenicity of Nyaditum Resae庐 Probiotic to Protect From Tuberculosis | Completed | NCT02076139 |
| 156 | Low Dose IL-2 for Ulcerative Colitis | Recruiting | NCT02200445 |
| 157 | Low-dose rhIL-2 in Patients With Recently-diagnosed Type 1 Diabetes | Recruiting | NCT02411253 |
| 158 | The Adoptive Immunotherapy for Solid Tumors Using Modified Autologous Cytokine-induced Killer Cells | Recruiting | NCT01868490 |
| 159 | Can Immune Parameters Predict Acute and Chronic Rejection in Lung Recipients? | Completed | NCT00340951 |
| 160 | Oral Tolerance in Cow鈥檚 Milk Allergy in the Infant | Unknown status | NCT00298376 |
| 161 | Search for a Link Between Response to Treatment and Circulating Leucocytes in High Grade Glioma Patients | Completed | NCT01836536 |
| 162 | Safety and Efficacy Study of Islets Xenotransplantation | Enrolling by invitation | NCT03162237 |
| 163 | Calorie Restriction in Multiple Sclerosis | Not yet recruiting | NCT04042415 |
| 164 | Safety, Tolerability, PK, PD, and Immunogenicity of Single and Multiple Ascending Intravenous Doses of FR104 | Completed | NCT02800811 |
| 165 | T Cell Effector and Regulatory Mechanisms in Asthma | Completed | NCT01612936 |
| 166 | Daily IL-2 for Steroid-Refractory Chronic Graft-versus-Host-Disease | Active, not recruiting | NCT01366092 |
| 167 | Immuno-regulatory Profiling of T Cells in GVHD Treated With Extracorporeal Photopheresis | Recruiting | NCT03851601 |
| 168 | The Immune System in End Stage CKD Patients - Comparison Among Different Modalities of RRT | Recruiting | NCT04286477 |
| 169 | The Effects of Prebiotics on Gut Bacterial Parameters, Immune Function & Exercise-Induced Airway Inflammation. | Recruiting | NCT02872675 |
| 170 | Effect of Specific Immunotherapy to Dust Mites in Children With Asthma | Unknown status | NCT00496561 |
| 171 | Impact of HIV Infection on Latent Tuberculosis (TB) Among Patients With HIV-TB Co-infection | Unknown status | NCT00692809 |
| 172 | Regulation of Mucosal Immune Response to Systemic MenB Vaccine | Completed | NCT00774384 |
| 173 | Hookworm Immune Regulation Project | Active, not recruiting | NCT02262403 |
| 174 | Low-dose Interleukin-2 for Treatment of Systemic Lupus Erythematosus | Recruiting | NCT03312335 |
| 175 | A Preliminary Study About Unexplained Recurrent Miscarriage and Repeated Implantation Failure Patients Treated With Low-dose Lymphocyte Immunotherapy | Completed | NCT03081325 |
| 176 | Epigenetic Regulation of Altered T-cell Immunity in Sarcoidosis | Recruiting | NCT03145922 |
| 177 | Ultra Low Dose Interleukin-2 in Healthy Volunteers | Completed | NCT01445561 |
| 178 | RFT-5-dgA in Patients With Metastatic Melanoma | Completed | NCT00314093 |
| 179 | Impact of Antimalarial Treatment on Measures of T Cell Suppression/Regulation in Healthy Adults From Doneguebougou, Mali | Completed | NCT02659566 |
| 180 | Study of the Frequency and of the Regulatory Function of Positive T Lymphocytes Dual CD4CD8aa (DP8a) Specific to a Bacteria of the Intestinal Microbiota (Faecalibacterium Prausnitzii) in Atopic Dermatitis, Asthma and Allergic Rhinitis | Unknown status | NCT02908360 |
| 181 | Effects of Plant Stanols on Immune Function in Asthma Patients | Completed | NCT01715675 |
| 182 | Immune Failure in Critical Therapy (INFECT) Study | Completed | NCT02186522 |
| 183 | Lactobacillus Reuteri Versus Placebo in the Treatment and Prevention of Infantile Colic | Recruiting | NCT00893711 |
| 184 | Bridging Pediatric and Adult Biomarkers in Graft-Versus-Host Disease | Active, not recruiting | NCT02194439 |
| 185 | Combination Daclizumab/Denileukin Diftitox to Treat Uveitis | Completed | NCT00326508 |
| 186 | Safety and Efficacy of Listeria in Combination With Chemotherapy as Front-line Treatment for Malignant Pleural Mesothelioma | Completed | NCT01675765 |
| 187 | Safety and Efficacy of Combination Listeria/GVAX Immunotherapy in Pancreatic Cancer | Completed | NCT01417000 |
| 188 | Monocyte Chemotactic Protein-1 (MCP-1) Expressing Monocytes to Predict Preterm Delivery: PhenoMAP Study | Completed | NCT01340222 |
| 189 | A Study of ZYC300 Administered With Cyclophosphamide Pre-Dosing | Completed | NCT00381173 |
| 190 | Postoperative REcurrence and DynamICs of T Cell Subsets in Crohn's Disease | Completed | NCT02770495 |
| 191 | The Mechanism of Enhancing the Anti-tumor Effects of CAR-T on PC by Gut Microbiota Regulation | Recruiting | NCT04203459 |
| 192 | Cytokine Regulation of Natural Killer Receptors in Inhibiting Activated T Cell Function | Unknown status | NCT00173290 |
